# Tissue-Specific Transcriptomic Insights into Myxozoan Infections: Immune and Stress Responses in the Kidney and Head Cartilage of Rainbow Trout

**DOI:** 10.3390/ijms27167253

**Published:** 2026-08-14

**Authors:** Naveed Akram, Reinhard Ertl, Reza Ghanei-Motlagh, Christopher J. Secombes, Mansour El-Matbouli, Mona Saleh

**Affiliations:** 1 Division of Fish Health, University of Veterinary Medicine, 1210 Vienna, Austria; naveed.akram@vetmeduni.ac.at (N.A.); rghanei-motlagh@upei.ca (R.G.-M.); mansour.el-matbouli@vetmeduni.ac.at (M.E.-M.); 2 VetCore, University of Veterinary Medicine, 1210 Vienna, Austria; reinhard.ertl@vetmeduni.ac.at; 3 Hoplite Research Lab, Department of Pathology and Microbiology, Atlantic Veterinary College, University of Prince Edward Island, Charlottetown, PE C1A 4P3, Canada; 4 Huntsman Marine Science Centre, St. Andrews, NB E5B 2L7, Canada; 5 Scottish Fish Immunology Research Centre, School of Biological Sciences, University of Aberdeen, Aberdeen AB24 3FX, UK; c.secombes@abdn.ac.uk

**Keywords:** proliferative kidney disease, transcriptomic analysis, immune pathways, target site

## Abstract

By damaging target tissues and compromising the host immune system, *Myxobolus cerebralis* and *Tetracapsuloides bryosalmonae* remain persistent threats to salmonids. RNA sequencing was used to assess transcriptome modulation in the kidney and head cartilage (HC) of rainbow trout during single and co-infections with these two myxozoan parasites. Fish were exposed to *M. cerebralis* (Mc) and *T. bryosalmonae* (Tb), and 30 days later, half of the fish from each group were subjected to sequential co-infections (Mc^+^ and Tb^+^). This assessment aimed to evaluate the combined effects of both pathogens. Transcriptomic analysis was conducted using kidney and HC tissues collected 60 days post-co-infection. The results showed that infection order altered host transcriptional profiles in a tissue-dependent manner. In the kidney, Tb fish showed pronounced transcriptional alterations linked to chronic inflammation, immune complex clearance, and IL-8-mediated responses. Tb^+^ fish exhibited broad immune activation in the kidney, suggesting dysregulated inflammatory responses likely associated with subsequent infection with *M. cerebralis*. In the kidney of Mc^+^ fish, the immune response was characterized by macrophage and IL signaling pathways. In HC, Mc fish activated Th17, IL-23, phagolysosome, and IL-6 cytokine pathways. In contrast, Mc^+^ fish showed increased pathogen processing and enhanced metabolic responses accompanied by suppression of cytokines and tissue repair processes. HC responses in Tb^+^ fish shifted toward IL-23, Th17-driven responses, alongside reduced developmental and extracellular matrix processes. The study establishes that host responses to myxozoan co-infection are shaped by tissue tropism and infection sequence, with distinct immune and tissue remodeling pathways activated in the target organs. These findings enhance the understanding of sequential *T. bryosalmonae* and *M. cerebralis* infections and provide a basis for identifying tissue-specific biomarkers and key immune targets. The findings may help identify methods for improved monitoring and management of myxozoan infections in salmonids.

## 1. Introduction

Parasites often exist in a dynamic equilibrium with their hosts, which can be disrupted by environmental changes, leading to disease outbreaks. They may induce mechanical damage such as tissue replacement, gill lamellae fusion, physiological alterations, impaired body condition, behavioral changes, and reduced reproductive capacity in fish [1,2,3]. Myxozoan parasites are major etiological agents of infectious diseases in salmonids, with *Myxobolus cerebralis* and *Tetracapsuloides bryosalmonae* representing two of the most impactful pathogens, causing whirling disease (WD) and proliferative kidney disease (PKD), respectively [4,5,6]. Both parasites are important for farmed and wild fish populations across Europe and North America [7]. *M. cerebralis*, a member of the class Myxosporea, infects the cartilaginous tissues of juvenile fish, where it disrupts skeletal development and causes the characteristic “whirling” swimming behavior accompanied by tail darkening or blackening [8]. Whereas *T. bryosalmonae* primarily targets the kidney, inducing chronic lymphoid hyperplasia and severe renal pathology in salmonids [9].

Co-infections frequently occur in natural aquatic ecosystems [10]; natural co-infection with these two specific parasites has not been fully characterized under natural conditions. Parasite co-infections are common in fish and can influence susceptibility, alter immune responses, and modify the progression and consequence of disease [11,12]. The interaction between co-infecting parasites can result in synergistic or antagonistic effects on host immune responses, leading to altered disease outcomes [13]. In teleosts, these interactions may be mediated by immunological mechanisms (cytokine signaling, inflammatory regulation, complement activity, and antigen processing and presentation) [14]. Depending on the sequence of infections, the primary parasite may either enhance or suppress these immune pathways, thereby altering host susceptibility to subsequent infections. Co-infecting pathogens may also face three different types of competition: apparent competition, interference competition, or resource competition [15]. Competition for host tissues and resources can lead to either synergistic or antagonistic effects, resulting from competitive exclusion or immune-driven facilitation [16]. Mixed infection with *Nucleospora cyclopteri* and *Kudoa islandica* in lumpfish has been linked to elevated mortalities [17]. Similarly, co-infection of *T. bryosalmonae* with *Raphidascaris acus* in wild brown trout (*Salmo trutta*) has been shown to impair recovery, suggesting a synergistic interaction that enhances parasite persistence through interference with the host immune response [18]. The interaction between *T. bryosalmonae* and *M. cerebralis* in rainbow trout has been studied in sequential co-infection models. When *M. cerebralis* infection preceded *T. bryosalmonae*, synergistic effects were observed, resulting in increased disease severity. In contrast, prior infection with *T. bryosalmonae* led to reduced clinical signs of whirling disease, though mortality rates remained unaffected [13]. Although the infection order influences the pathological outcomes of co-infections, the specific molecular mechanisms in the primary target tissues remain poorly understood. This study employs genome-wide RNA sequencing to analyze host responses in the kidney and head cartilage, aiming to identify immune, metabolic, and tissue-remodeling pathways affected by parasite tropism and the sequence of infection.

The application of high-throughput transcriptomic technologies, such as RNA sequencing, provides powerful tools for understanding host–pathogen interactions at the molecular level through detailed analysis of gene expression [19]. RNA-seq enables comprehensive quantification of transcripts linked to specific physiological or pathological conditions, providing improved resolution over hybridization-based approaches such as microarrays. This method has facilitated significant progress in mapping and annotating transcriptomes and in uncovering novel transcripts and isoforms across diverse fish species, including those lacking reference genomes [20]. Transcriptomic profiling has emerged as a key tool in fish immunology for understanding host responses to parasite, viral, and bacterial infections [21,22]. RNA sequencing analyses have identified genes involved in important immunological processes, including NOD-like receptors, chemokines, cytokine signaling, antigen presentation, complement activation, pattern recognition, and cellular adhesion [23,24]. These results have been documented in many species important to aquaculture, advancing our knowledge of the molecular processes driving both innate and adaptive immune responses [25]. Experimental co-infection studies in rainbow trout have demonstrated modulated expression of immune-related genes such as suppressors of cytokine signaling (SOCS) and components of the JAK-STAT signaling pathway, suggesting potential immunological interference between *M. cerebralis* and *T. bryosalmonae* during host immune activation [14].

In the present study, we employed transcriptomic profiling to investigate the immune response of rainbow trout (*Oncorhynchus mykiss*) to single and co-infection with *M. cerebralis* and *T. bryosalmonae*. To investigate tissue-specific host responses across various infectious groups, we focused on two primary target sites of parasite development: the head cartilage, which is infected by *M. cerebralis* [6], and the kidney, which is the primary site of infection for *T. bryosalmonae* [26]. This study aims to identify gene tissue-specific transcriptional expression profiles associated with single and sequential co-infections with *M. cerebralis* and *T. bryosalmonae*, providing insight into how parasite tropism and infection order modulate host transcriptional responses in the kidney and HC of rainbow trout.

## 2. Results

### 2.1. Parasite Load

**Figure 1:**
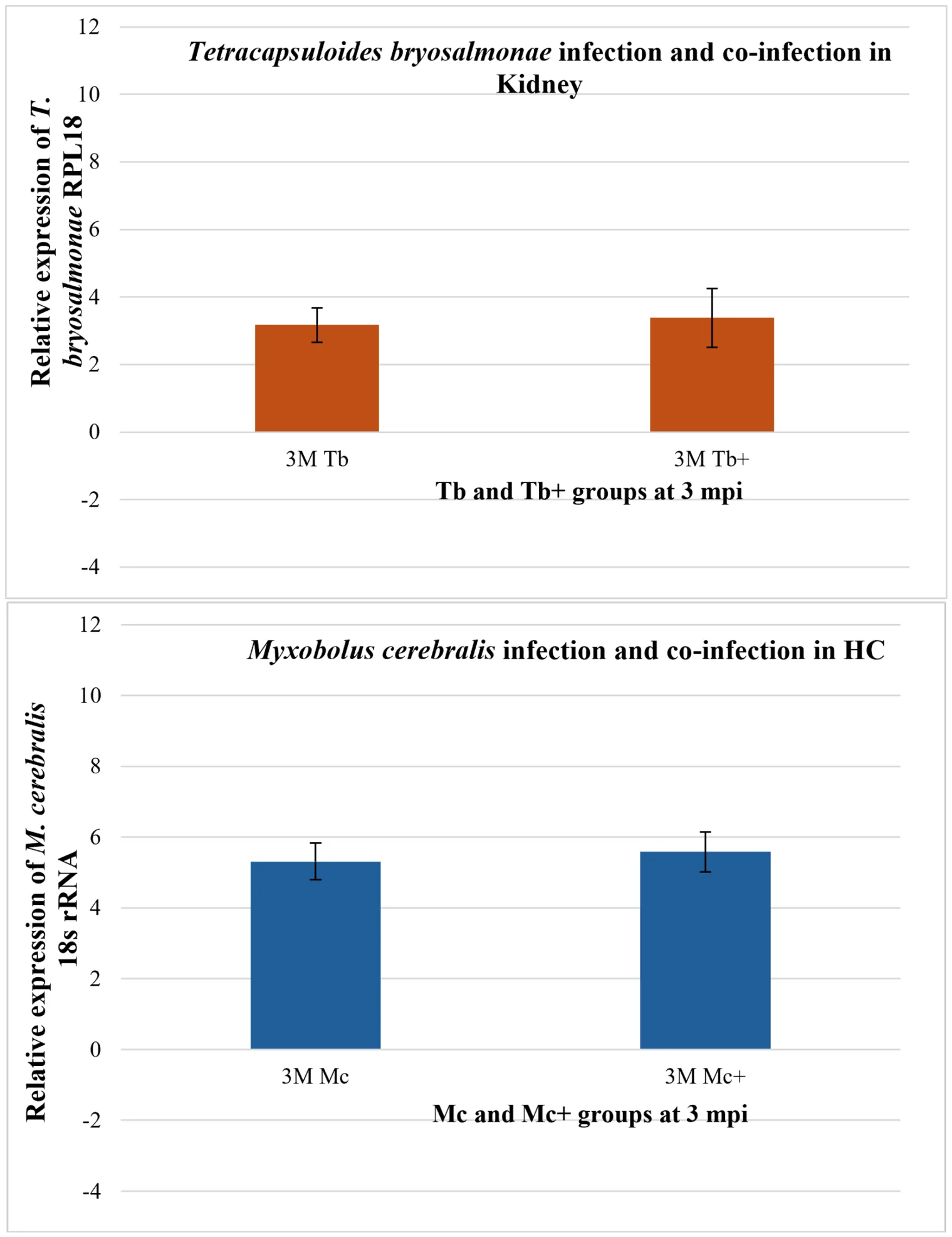
Relative expression of *T. bryosalmonae* *RPL18* in the kidneys (*p* = 0.84) and *M. cerebralis* *18s rRNA* in the head cartilage (*p* = 0.73) of single and co-infected trout at 3 months (3M) post-infection. Bars represent mean ± SEM.

Parasitic load assessed by qPCR showed comparable *M. cerebralis* 18S rRNA levels in HC of Mc and Mc^+^ and *T. bryosalmonae* RPL18 levels in the kidney of Tb and Tb^+^ trout at 3 months post-infection (Figure 1). Despite relative similarity of parasitic burden, transcriptomic responses were different among groups in both organs.

### 2.2. Differential Expression Analysis in Kidney

**Figure 2:**
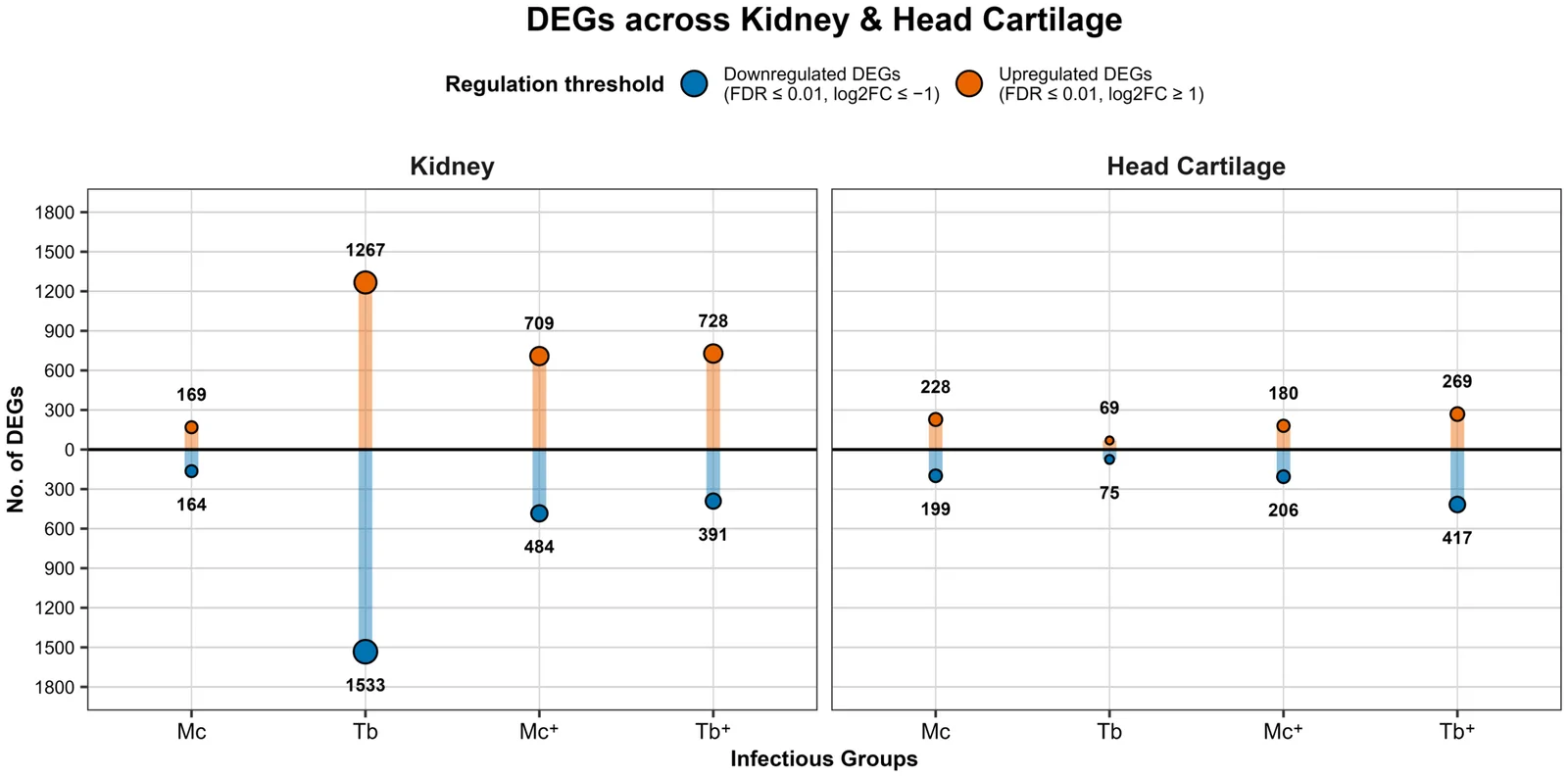
Bidirectional bubble plot of upregulated and downregulated DEGs in kidney and head cartilage of rainbow trout (infected vs. control).

**Figure 3:**
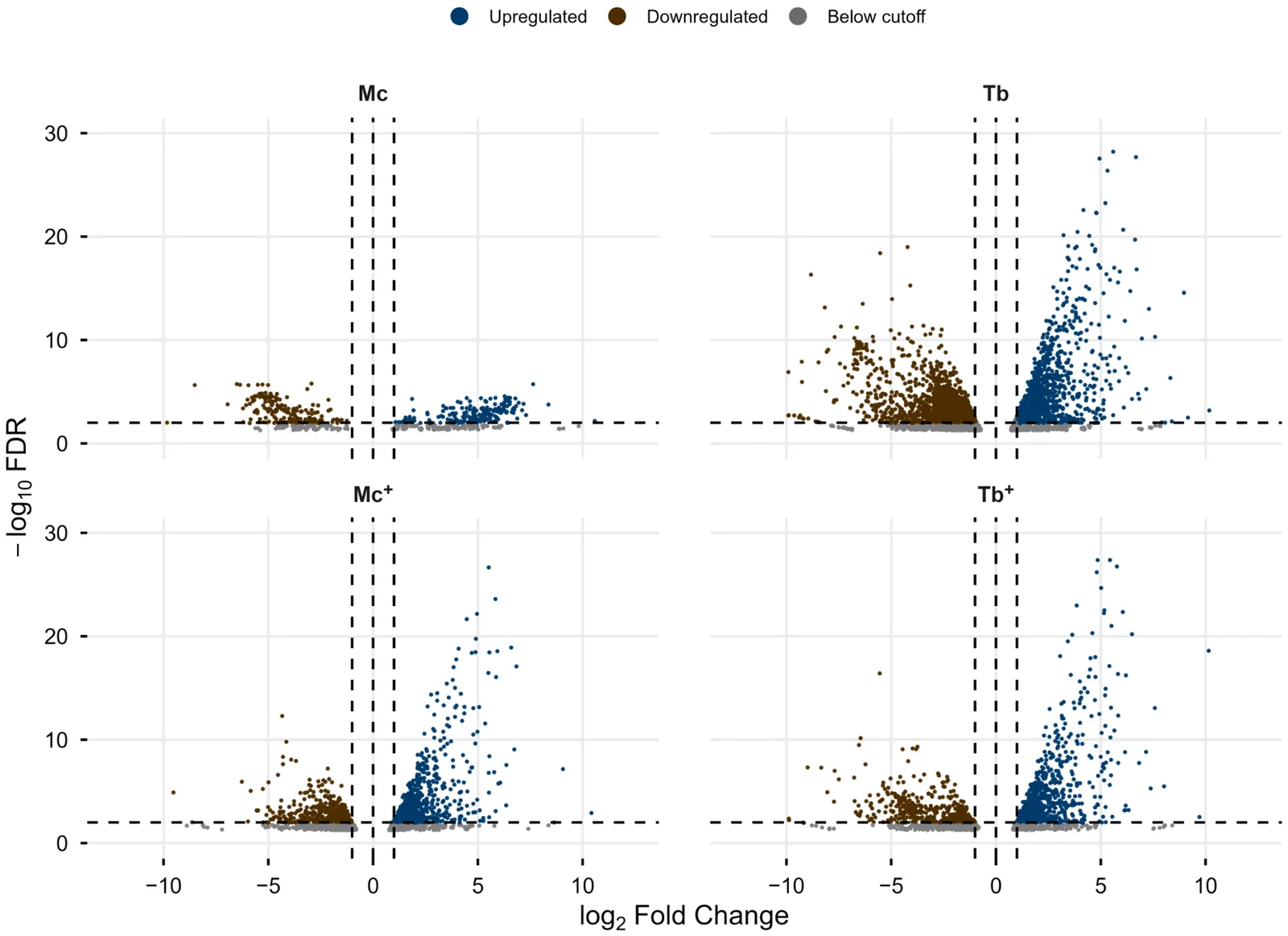
Volcano plots visualizing DEGs in the kidney across the four experimental groups.

A total of approximately 935 million high-quality paired-end reads were retained after trimming across all 15 posterior kidney RNA-seq libraries, with average read lengths ranging from 121.17 to 126.08 base pairs. Post-trimming, each library contributed between 52.55 and 72.99 million reads (Supplementary File S1). Transcript expression analysis of singly infected and co-infected rainbow trout groups revealed multiple differentially expressed genes (DEGs) in the kidney (log_2_ fold change > 1, FDR *p*-value ≤ 0.01). DEG counts reflected tissue tropism: Tb showed the strongest response (2800 DEGs) in the kidney (its target organ), while Mc was modest (333 DEGs) due to parasite absence from this tissue. Both co-infection groups, Mc^+^ (1193) and Tb^+^ (1119), showed increased transcriptomic responses (Figure 2; Supplementary File S2). The volcano plot (Figure 3) highlights a substantial number of DEGs expressed across the various infection groups in the kidney.

### 2.3. Differential Expression Analysis in Head Cartilage (HC)

**Figure 4:**
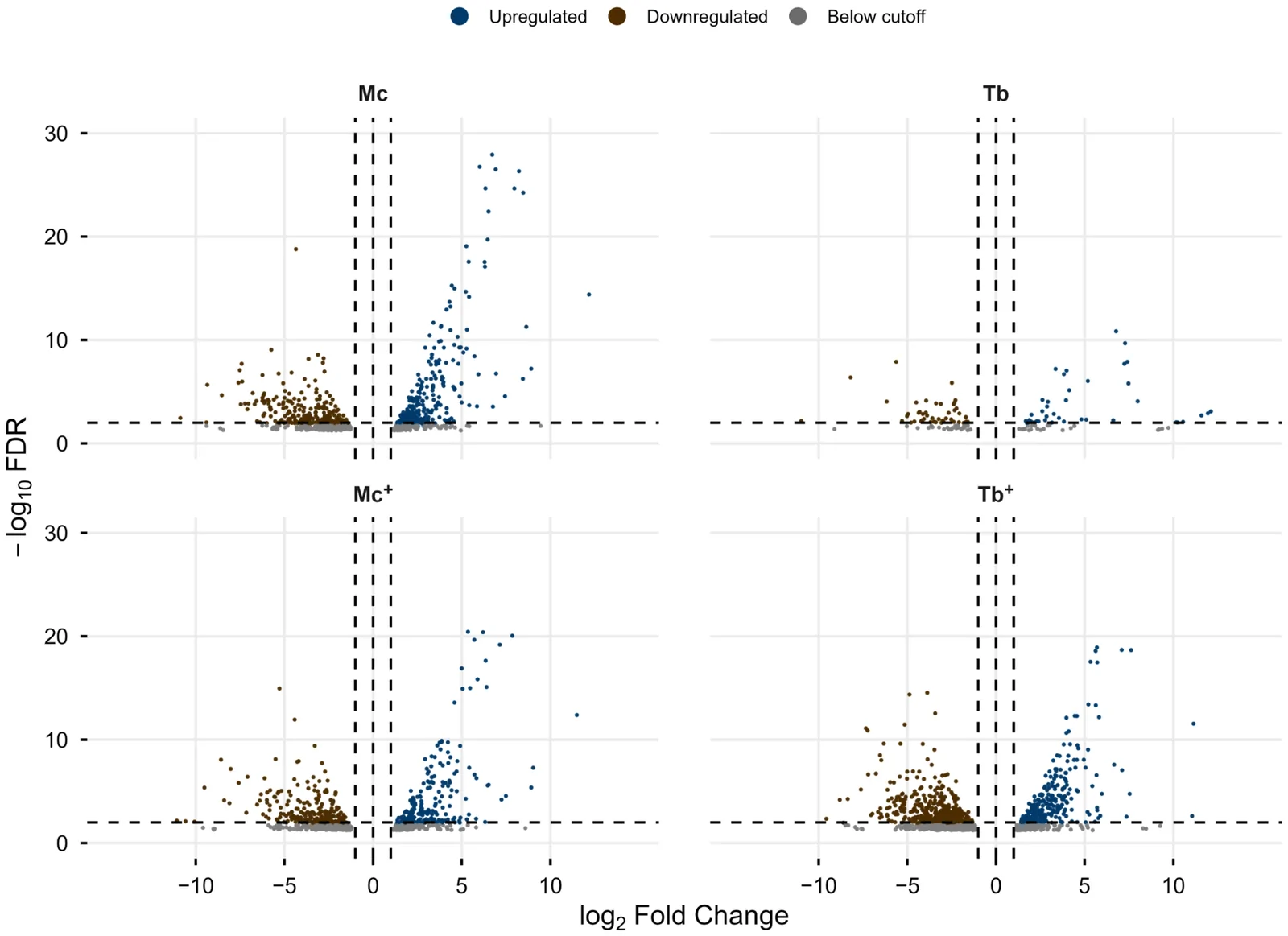
Volcano plots visualizing DEGs in the head cartilage across the four experimental groups.

A total of approximately 1.24 billion high-quality paired-end reads were retained after trimming across all 15 HC RNA-seq libraries, with average read lengths ranging from 100.85 to 117.86 base pairs. Post-trimming, each library contributed between 69.34 and 96.03 million reads (Supplementary File S1). Transcript expression analysis of single-infected and co-infected rainbow trout groups revealed various differentially expressed genes (DEGs) in HC (log_2_ fold change > 1, FDR *p*-value ≤ 0.01). Head cartilage, being a primary target site for *M. cerebralis*, showed an overall lesser transcriptome response; Tb^+^ showed a significantly stronger reaction (686 DEGs), Mc and Mc^+^ were intermediate (427 and 386, respectively), and Tb was minimal with 144 DEGs, likely due to the tissue not being its site of infection (Figure 2; Supplementary File S3). The volcano plot (Figure 4) highlights a substantial number of DEGs expressed across the various infection groups in the head cartilage tissue.

**Figure 5:**
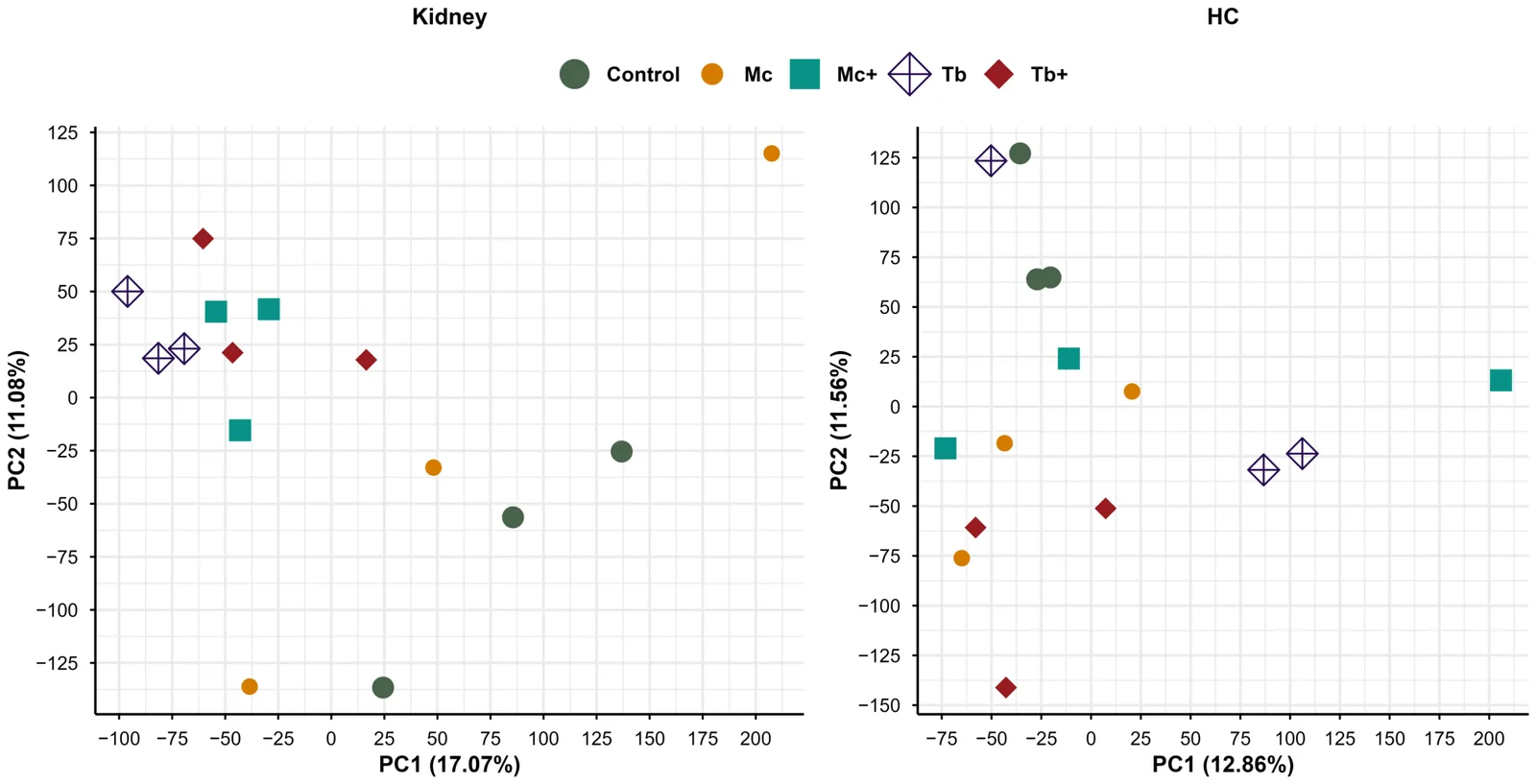
Principal component analysis (PCA) of gene expression profiles in kidney (**left**) and head cartilage (HC) (**right**) based on counts per million (CPM) values.

In the kidney, PC1 (17.07%) separated controls from infected samples, with Tb, Mc^+^, and Tb^+^ forming distinct clusters, and Mc appearing more dispersed (Figure 5). In head cartilage, PC1 (12.86%) placed Mc and Tb^+^ in close proximity, Mc^+^ was moderately offset, and Tb formed a distinct cluster (Figure 5).

### 2.4. Shared and Unique DEGs in Kidney

**Figure 6:**
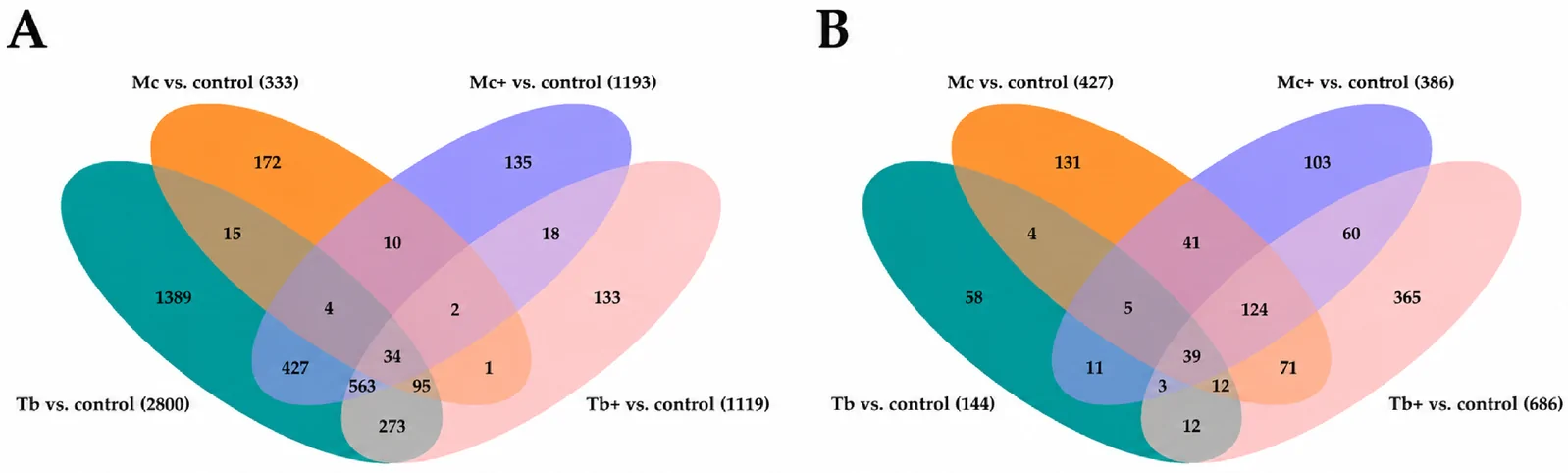
The Venn diagram illustrates the identified genes in the kidney (**A**) and head cartilage (**B**) across all groups.

Transcriptional similarities and differences among experimental groups in the kidney revealed 34 DEGs shared across all four infection conditions (Mc, Mc^+^, Tb, and Tb^+^). The Mc group exhibited 172 unique DEGs, and Mc^+^ exhibited 135. The Tb group exhibited the highest 1389 number of uniquely differentially expressed genes and Tb^+^ exhibited 133. Additional pairwise and three-group overlaps are shown in Figure 6A.

### 2.5. Shared and Unique DEGs in HC

In HC, a total of 39 DEGs were commonly regulated across all four infection groups (Mc, Mc^+^, Tb, and Tb^+^), reflecting a shared transcriptional response. Among unique DEGs, the Mc group expressed 131, and Mc^+^ expressed 103 DEGs. The Tb group had 58, and the Tb^+^ group showed the highest number, 365. Additional pairwise and three-group overlaps are shown in Figure 6B.

### 2.6. Gene Ontology (GO) of the DEGs in the Kidney

#### 2.6.1. Upregulated Genes in the *M. cerebralis*-Infected (Mc) and Sequential Co-Infection (Mc^+^) Group

Mc exhibited increased expression of *POMZP3* transcripts, *pom121*, and zona pellucida glycoprotein 3, with fold-change values of 329.93 and 107.17, respectively. Other highly expressed genes included *eif4e1b* (113.29), *h2bk1* (104.97), and *foxr2* (7.18), whereas Mc^+^ showed strong induction of caspase recruitment domain family member 18, *card18*, with a fold change of 67.12, PR/SET domain 1, *prdm1*, with 57.62, transmembrane serine protease 13 *tmprss13*, with 54.76, interleukin 10, *il10*, with 46.88; and TNF receptor superfamily member 13B, *tnfrsf13b*, with 46.35.

**Figure 7:**
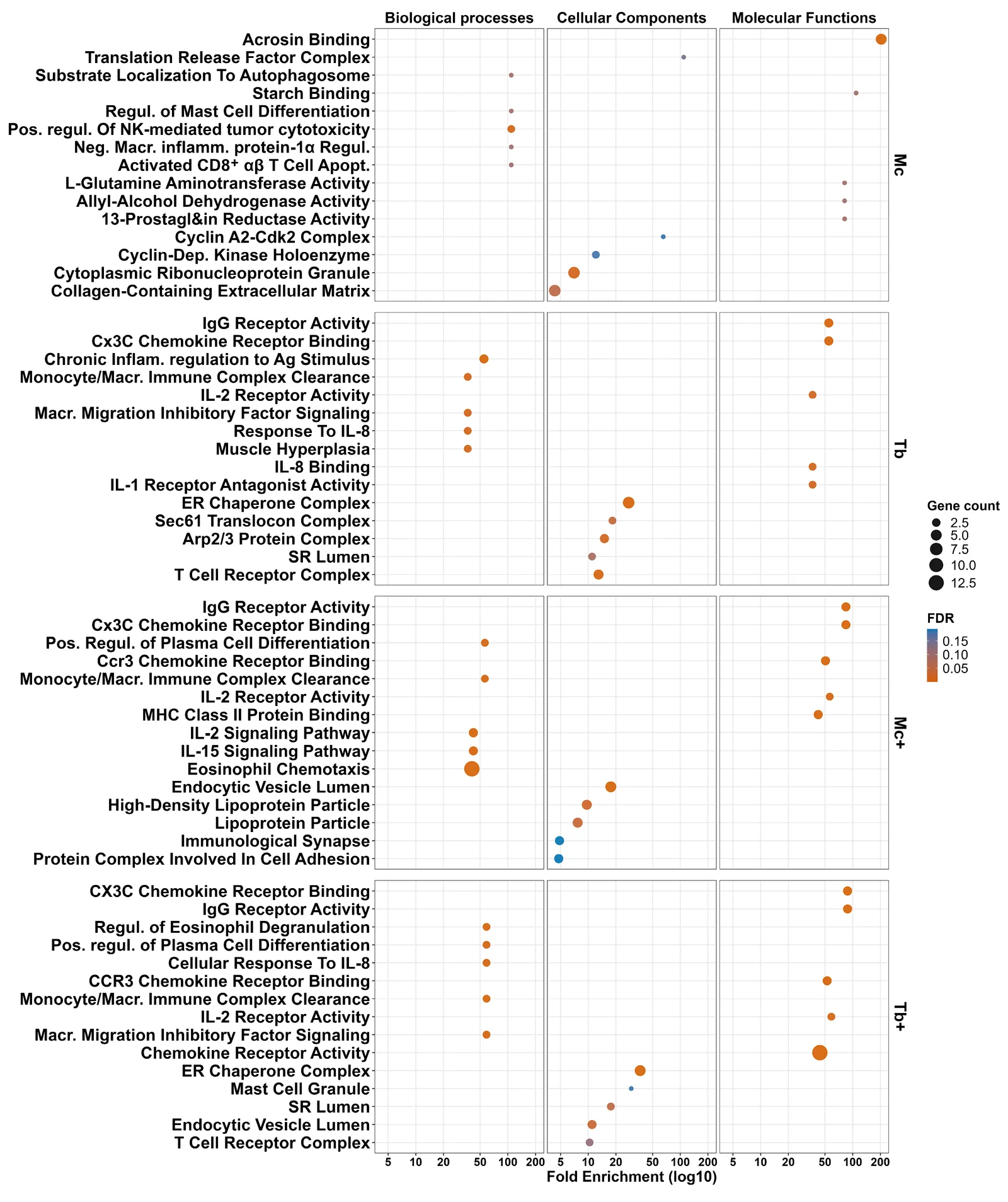
Upregulated Gene Ontology terms along with fold enrichment in the kidney across the four groups.

GO enrichment of upregulated genes in the Mc group pointed to translational control and cell-cycle machinery (translation release factor complex, cyclin A2-CDK2, related CDK holoenzymes) and RNA granules, with additional links to the collagen-containing extracellular matrix. Immune-related terms were oriented towards regulation rather than activation, including modulation of NK-cell cytotoxicity, mast-cell differentiation, autophagic trafficking, reduced MIP-1α production, and apoptosis of activated CD8^+^ T cells. In the Mc^+^ group, enrichment was observed for immune complex clearance by monocytes and macrophages, teleost Fc-receptor analogs, MHC II binding, the immunological synapse, and *IL-2* and *IL-15* signaling pathways. CCR3 and CXC3 chemokine receptor binding and eosinophilic chemotaxis. Cellular localizations in endocytic vesicle lumen, lipoprotein, HDL particles, and cell adhesion complexes suggest active antigen presentation and immune cell transcriptomic coordination. Enrichment details of pathways related to biological processes, cellular components, and molecular functions are shown in Figure 7.

#### 2.6.2. Upregulated Genes in the *T. bryosalmonae*-Infected (Tb) and Sequential Co-Infection (Tb^+^) Group

The single infected group (Tb) exhibited significant upregulation of TNF receptor superfamily member 13B, *tnfrsf13b*, with a fold change of 193.10, followed by PR/SET domain 1, *prdm1*, with 110.02, caspase recruitment domain family member 18, *card18*, with 79.48, TNF receptor superfamily member 6b, *tnfrsf6b*, with 75.75; and aconitate decarboxylase 1, *acod1*, with 70.81. In the Tb^+^ group, the most prominently upregulated genes included TNF receptor superfamily member 13B, tnfrsf13b, with a fold change of 142.57, caspase recruitment domain family member 18, *card18*, with 113.81, PR/SET domain 1, *prdm1*, with 72.84; TNF receptor superfamily member 6b, *tnfrsf6b*, with 54.62; and *CX3C* motif chemokine ligand 1, *cx3cl1*, with 43.35.

Compared with the Tb group, sequential infection (Tb^+^) retained the core immune features observed during *T. bryosalmonae* infection while strengthening selected adaptive immune functions. In the Tb group, enriched BP terms included regulation of chronic inflammation and immune complex clearance, with chemokine-driven recruitment, response to *IL-8*, and macrophage migration inhibitory factor (MIF), and the muscle hyperplasia term likely reflected tissue remodeling. Following sequential infection (Tb^+^), these responses were maintained, with continued enrichment of immune-complex clearance, MIF signaling, and IL-8-mediated recruitment, together with enhanced eosinophil degranulation and plasma cell differentiation. Cellular component analysis in both groups mainly involved secretory and vesicular compartments, including the endoplasmic reticulum and sarcoplasmic reticulum lumens, whereas Tb^+^ additionally showed enrichment of the endocytic vesicle lumen, the immunological synapse through the T-cell receptor complex, and mast cell granules. Similarly, molecular function terms in both groups highlighted Fc receptor activity, IL-2 receptor signaling, and chemokine receptor interactions, while Tb^+^ further exhibited enrichment of IgG receptor activity and CCR3 chemokine receptor binding (Figure 7).

#### 2.6.3. Downregulated Genes in the *M. cerebralis*-Infected (Mc) and Sequential Co-Infection (Mc^+^) Group

In the Mc group, markedly downregulated genes included cystatin C, *cst3*, with a fold change of −122.99; apolipoprotein B, *apob*, with −90.53; chromosome 10 open reading frame 71, *C10orf71*, with −81.31; troponin T3, fast skeletal type, *tnnt3*, with −74.08; and ATP binding cassette subfamily B member 11, *abcb11*, with −73.36. In the Mc^+^ group, strong downregulation was evident for cystatin C, *cst3*, with a fold change of −741.38, followed by PZP alpha-2-macroglobulin-like, *pzp*, with −62.54; chromosome 6 open reading frame 58, *c6orf58*, with −57.35; CD209 molecule, *cd209*, with −47.08; and apolipoprotein B, *apob*, with −38.33.

**Figure 8:**
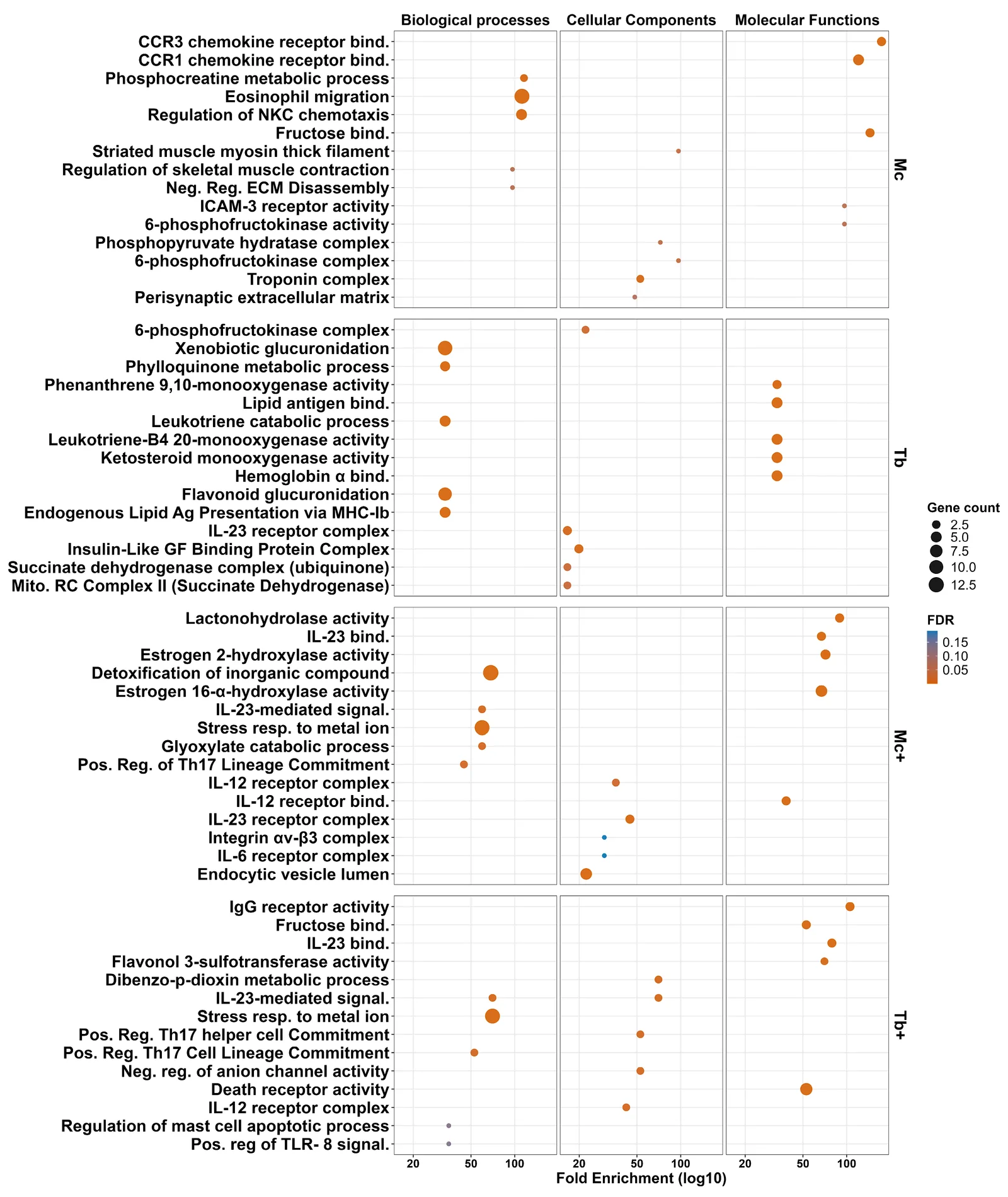
Downregulated Gene Ontology terms along with fold enrichment in the kidney across the four groups.

In Mc fish, downregulated pathways were mainly associated with energy metabolism and chemokine-guided trafficking, including the 6-phosphofructokinase complex and phosphopyruvate hydratase complex. Suppression was also observed for chemokine axes linked to CCR1, CCR3, and NK-cell chemotaxis, and ICAM-3 receptor activity. Genes typically involved in limiting ECM breakdown, along with muscle-structure genes such as myosin thick filament, troponin, and presynaptic ECM components, are also reduced (Figure 8). In contrast, Mc^+^ showed decreased enrichment of pathways for detoxification of inorganic compounds, metal-ion stress response, and glyxolate catabolism accompanied by suppressed *IL-6*, *IL-12*, and *IL-23* pathways. Reduced adhesion and vesicular uptake were associated with lower integrin alpha v-beta 3 complex and endocytic vesicle lumen. Molecular functions reflected decreased *IL-23* binding, *IL-12* receptor binding, and specific hydrolase and estrogen-hydroxylase activities (Figure 8).

#### 2.6.4. Downregulated Genes in the *T. bryosalmonae*-Infected (Tb) and Sequential Co-Infection (Tb^+^) Group

The kidney of the Tb group exhibited downregulation of several genes, including secreted phosphoprotein 2, *spp2*, with a fold change of −948.27; chromosome 10 open reading frame 71, *c10orf71*, with −648.46; complement factor B, *cfb*, with −599.12; hyaluronan-binding protein 2, *habp2*, with −498.22; and afamin, *afm*, with −455.09. Similarly, in the Tb^+^ group, multiple genes were markedly downregulated such as chromosome 6 open reading frame 58, *c6orf58*, with a fold change of −950.21; secreted phosphoprotein 2, *spp2*, with −939.31; apolipoprotein B, *apob*, and PZP alpha-2-macroglobulin-like, *pzp*, with fold changes of −324.05 and −208.33, respectively; and alpha-2-macroglobulin-like 1, *a2ml1*, with −180.89.

In Tb fish, downregulated GO terms mainly related to detoxification and energy processes, such as xenobiotic and flavonoid glucuronidation, leukotriene breakdown, vitamin K pathways, the glycolytic 6-phosphofructokinase complex, and mitochondrial complex II. Additionally, there was reduced enrichment in pathways like lipid antigen presentation through MHC class Ib, IL-23 signaling, and the IGF-binding protein complex, along with decreases in lipid antigen binding, hemoglobin-α binding, and monooxygenase activities. In contrast, Tb^+^ mainly exhibited downregulation of immune-related pathways such as responses to metal-ion stress, IL-23 signaling, T-helper 17 cell lineage regulation, mast cell apoptosis control, and Toll-like receptor 8 signaling. Similarly, there was reduced enrichment in the IL-12 receptor complex, along with decreased IgG receptor activity, IL-23 binding, flavonol 3-sulfotransferase activity, death receptor functions, and fructose binding. (Figure 8). A detailed overview of the enriched pathways in the kidney and the associated gene counts is provided in Supplementary File S4.

#### 2.6.5. KEGG Pathway Analysis of Kidney DEGs

**Figure 9:**
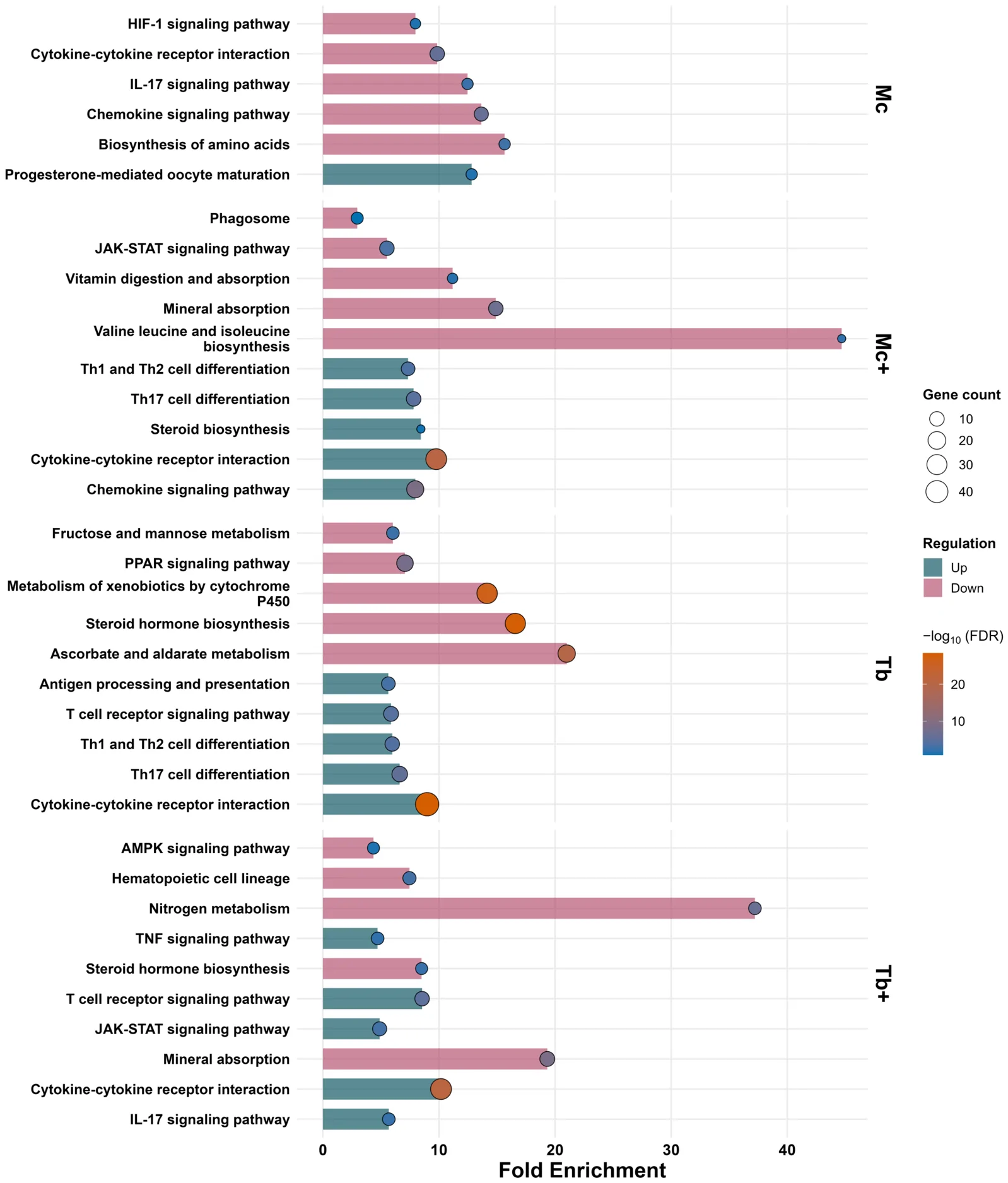
Significantly regulated KEGG pathways in infected kidney.

KEGG enrichment analysis revealed distinct pathway alterations across the different experimental groups (Supplementary File S5). In the *M. cerebralis* group, progesterone-mediated oocyte maturation was the only pathway enriched among upregulated genes, whereas amino acid biosynthesis, the chemokine signaling pathway, the IL-17 signaling pathway, cytokine-cytokine receptor interaction, and the HIF-1 signaling pathway were enriched among downregulated genes. In the Mc^+^ group, pathways enriched among upregulated genes included cytokine-cytokine receptor interaction, steroid biosynthesis, chemokine signaling pathway, Th17 cell differentiation, and Th1 and Th2 cell differentiation, whereas mineral absorption, JAK-STAT signaling pathway, phagosome, valine, leucine, and isoleucine biosynthesis, and vitamin digestion and absorption were enriched among downregulated genes. The *T. bryosalmonae* group showed upregulated enrichment of cytokine-cytokine receptor interaction, Th17 cell differentiation, Th1 and Th2 cell differentiation, T cell receptor signaling pathway, and antigen processing and presentation, while ascorbate and aldarate metabolism, steroid hormone biosynthesis, metabolism of xenobiotics by cytochrome P450, PPAR signaling pathway, and fructose and mannose metabolism were enriched among downregulated genes. In the Tb^+^ group, pathways enriched among upregulated genes comprised cytokine-cytokine receptor interaction, T cell receptor signaling pathway, *IL-17* signaling pathway, JAK-STAT signaling pathway, and *TNF* signaling pathway, while steroid hormone biosynthesis, hematopoietic cell lineage, AMPK signaling pathway, nitrogen metabolism, and mineral absorption were enriched among downregulated genes (Figure 9). Supplementary File S6 shows the enriched KEGG pathways in the kidney along with the number of identified genes.

### 2.7. Gene Ontology (GO) of the DEGs in the Head Cartilage (HC)

#### 2.7.1. Upregulated Genes in the *M. cerebralis*-Infected (Mc) and Sequential Co-Infection (Mc^+^) Group

In the Mc group, several genes were strongly regulated, including mannose receptor C-type 1, *mrc1*, with a fold change of 250.88; C-reactive protein, *crp*, with 122.39; matrix metallopeptidase 1, *mmp1*, with 121.43; hemopexin, *hpx*, with 79.34; and immunoglobulin heavy constant mu, *ighm*, with 77.99. Following sequential co-infection with *T. bryosalmonae*, phospholipase A2 group IVC, *pla2g4c*, with a fold change of 485.89; transglutaminase 5, *tgm5*, with 91.51; C-reactive protein, *crp*, with 88.11; immunoglobulin heavy constant mu, *ighm*, with 81.78; and matrix metallopeptidase 1, *mmp1*, with 73.42, were upregulated. Thus, *crp*, *ighm*, and *mmp1* were upregulated in both infection groups.

**Figure 10:**
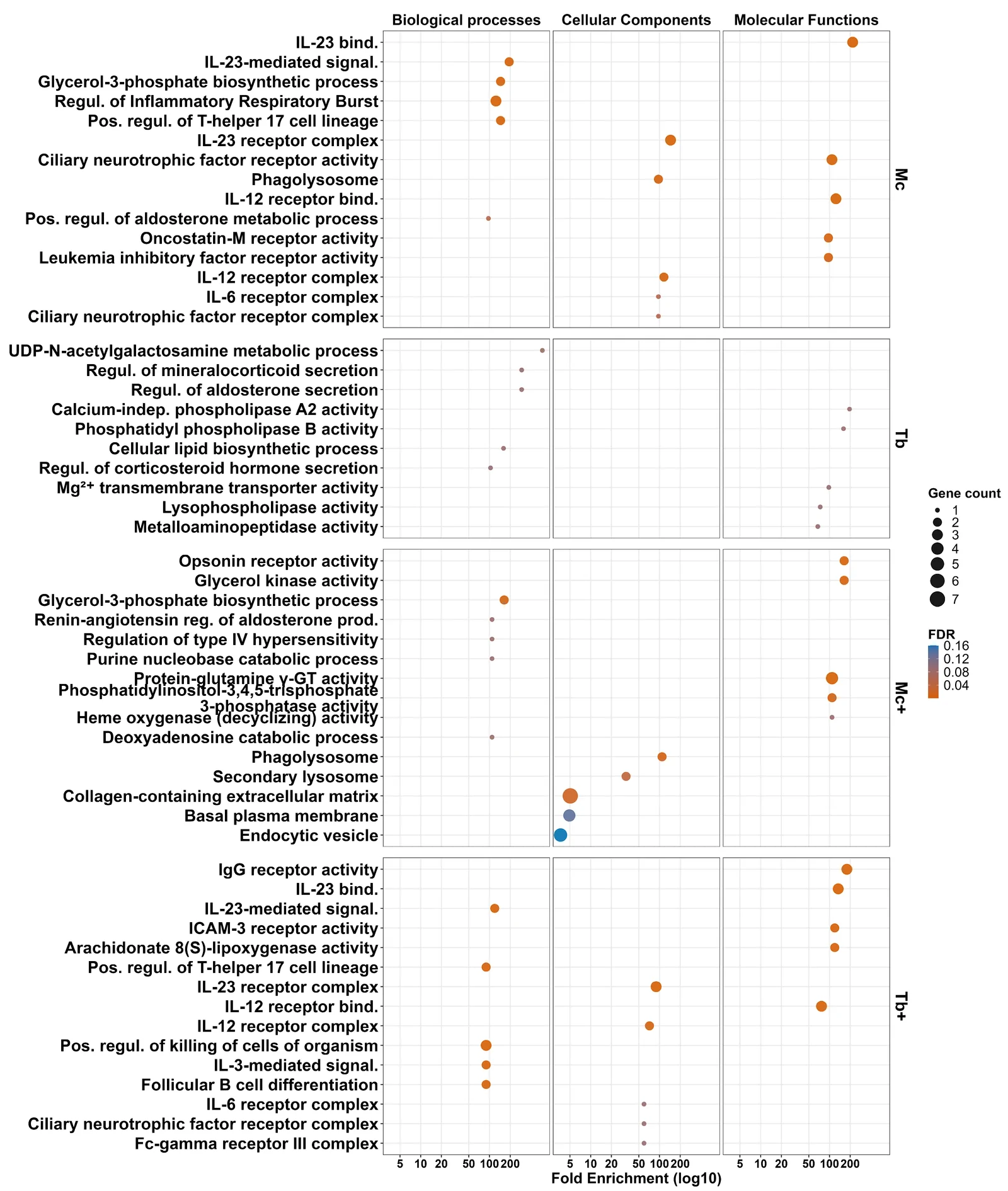
Upregulated Gene Ontology terms along with fold enrichment in head cartilage across the four groups.

In Mc fish, upregulated GO terms included IL-6, IL-12, and IL-23 receptor complexes and activities, together with respiratory burst and phagolysosome pathways. Glycerol-3-phosphate biosynthesis and the aldosterone metabolic process were also enriched. In contrast, Mc^+^ exhibited enhanced phagocytic activity, reflected by increased opsonin receptor activity and enrichment of phagolysosomes and secondary lysosomes. Lipid and membrane biogenesis was further supported by enrichment of glycerol-3-phosphate biosynthesis, glycerol kinase, endocytic vesicles, the basal plasma membrane, and the collagen-containing extracellular matrix. Additionally, Mc^+^ showed increased enrichment of type IV hypersensitivity and renin-angiotensin regulation of aldosterone, together with purine and deoxyadenosine catabolism, transglutaminase and heme oxygenase activities, and PI (3,4,5) P_3_ 3-phosphatase activity (Figure 10).

#### 2.7.2. Upregulated Genes in the *T. bryosalmonae*-Infected (Tb) and Sequential Co-Infection (Tb^+^) Group

The Tb group exhibited marked upregulation of Crystallin beta B1, *crybb1*, showing an exceptionally high fold change of 4465.38, while phospholipase A2 group IVC, *pla2g4c*, reached 97.95. Increased expression was observed for crystallin gamma N, *crygn*, with 62.71; a second *crybb1* transcript, with 43.66; and beta-1,4-N-acetyl-galactosaminyltransferase 2, *b4galnt2*, with 34.13. While in the Tb^+^ group, strong upregulation was observed for hemoglobin subunit epsilon 1, *hbe1*, with a fold change of 164.25, followed by C-reactive protein, *crp*, with 138.42. Mannose receptor C-type 1, *mrc1*, with 56.27; immunoglobulin heavy constant mu, *ighm*, with 49.39; and hemopexin, *hpx*, with 23.57, were also among upregulated genes.

Compared to the Tb group, the Tb^+^ group showed a shift from metabolic to immune-related pathways (Figure 10). In Tb, enriched biological processes involved UDP-N-acetylgalactosamine metabolism, cellular lipid biosynthesis, and the regulation of mineralocorticoid and aldosterone secretion. No significant enrichment was found in cellular component terms. The molecular function terms included activities such as calcium-independent phospholipase A2, phosphatidyl phospholipase B, lysophospholipase activity, magnesium transport, and metalloaminopeptidase activity. Conversely, Tb^+^ exhibited enrichment in immune pathways, such as IL-6, IL-12, and IL-23 signaling, indicating a Th17-type immune response. Cellular component terms related to IL-6, IL-12, IL-23, FcγRIII, and CNTF receptor complexes were enriched, along with additional pathways including ICAM-3-mediated adhesion, IL-3 signaling, and arachidonate 8(S)-lipoxygenase activity.

#### 2.7.3. Downregulated Genes in the *M. cerebralis*-Infected (Mc) and Sequential Co-Infection (Mc^+^) Group

In the Mc group, several genes were markedly downregulated. Tryptophan hydroxylase 1, *tph1*, appeared twice, with substantial fold-change reductions of −192.28 and −166.96, respectively. Other strongly suppressed genes included uromodulin-like 1, *umodl1*, with −110.49; paired box 8, *pax8*, with −95.64; and acetylserotonin O-methyltransferase, *asmt*, with −75.83. In the Mc^+^ group, downregulated genes included Potassium sodium-activated channel subfamily T member 2, *kcnt2*, which showed the highest fold reduction, at −337.52, followed by tryptophan hydroxylase 1, *tph1*, with −190.78. Additionally, pleckstrin homology and coiled-coil domain-containing D1, *plekhd1*, decreased by −142.51; leucine-rich repeat LGI family member 2, *lgi2*, by −135.23; and cystatin C, *cst3*, by −93.37.

**Figure 11:**
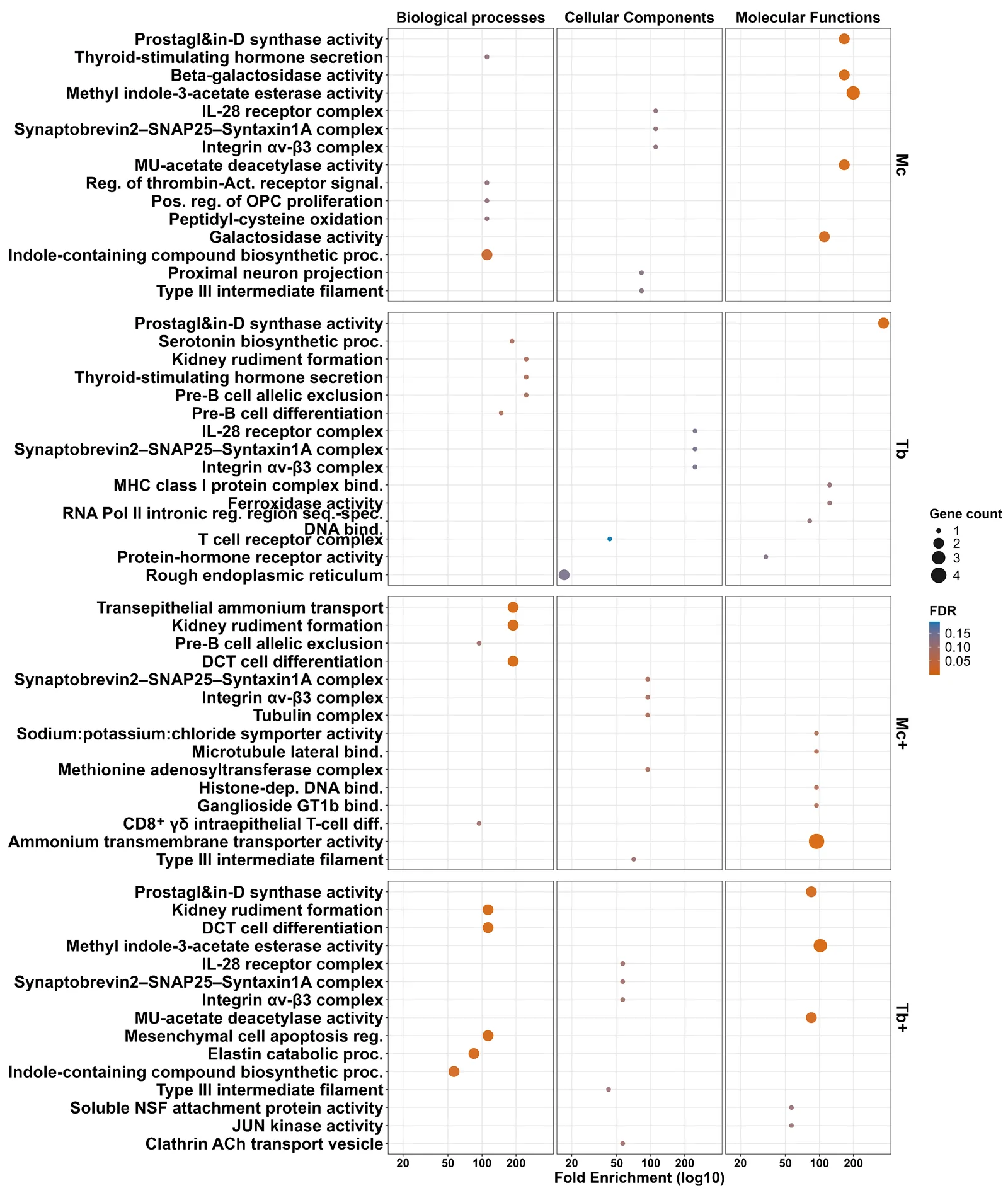
Downregulated Gene Ontology terms along with fold enrichment in head cartilage across the four groups.

Compared to the Mc group, the Mc^+^ group exhibited a shift in the downregulated pathways from metabolic and signaling processes towards epithelial transport, kidney development, and immune cell differentiation (Figure 11). In Mc, downregulated biological process terms included indole biosynthesis, peptidyl-cysteine oxidation, thyroid-stimulating hormone secretion, thrombin-activated receptor signaling, and oligodendrocyte progenitor proliferation. Cellular component terms were associated with receptor and adhesion machinery, vesicle fusion modules, and proximal neuron projections, including the integrin αvβ3 complex, the SNARE complex (synaptobrevin-2, SNAP-25, syntaxin-1A, and complexin I), type III intermediate filaments, and proximal neuron projections. Molecular function terms showed reduced glycosidase and esterase activities, along with lower prostaglandin-D synthase activity. In contrast, the Mc^+^ group was characterized by downregulation of biological processes related to transepithelial ammonium transport, kidney rudiment formation, distal convoluted tubule cell differentiation, CD8^+^ γδ intraepithelial T-cell differentiation, and pre-B-cell allelic exclusion. The downregulated cellular component terms included the integrin αvβ3 complex, tubulin and methionine adenosyltransferase complexes, the SNARE complex, and type III intermediate filaments, while molecular function terms reflected reduced ammonium transporter, Na, K, Cl symporter, histone-dependent DNA-binding, microtubule lateral-binding, and ganglioside GT1b-binding activities.

#### 2.7.4. Downregulated Genes in the *T. bryosalmonae*-Infected (Tb) and Sequential Co-Infection (Tb^+^) Group

The Tb group within HC showed a clear reduction in paired box 8 (Pax8), with a fold change of −177.74. Other genes also had decreased expression, including acetylserotonin O-methyltransferase, *asmt*, with −71.47; tryptophan hydroxylase 1, *tph1*, with −60.76; retinol dehydrogenase 8, *rdh8*, with −32.56; and ankyrin repeat domain 34B, *ankrd34b*, with −18.81. In the Tb^+^ group, several genes were significantly downregulated. Notably, Prostaglandin D2 synthase, *ptgds*, showed the strongest fold reduction, with −1622.05, followed by cystatin C, *cst3*, with −448.00; potassium sodium-activated channel subfamily T member 2, *kcnt2*, with −328.41; ceruloplasmin, *cp*, with −150.91; and leucine-rich repeat LGI family member 2, *lgi2*, with −147.80.

In Tb fish, biological process terms such as B-cell maturation, thyroid-stimulating hormone secretion, and serotonin biosynthesis were downregulated. Cellular component terms involved receptor and trafficking structures, including the integrin αvβ3 complex, the T-cell receptor complex, the SNARE complex (synaptobrevin-2, SNAP-25, syntaxin-1A, and complexin I), and the rough endoplasmic reticulum. Molecular function terms indicated decreased prostaglandin-D synthase and ferroxidase activities, along with reduced binding to MHC class I protein complexes, diminished sequence-specific DNA binding at RNA polymerase II intronic regulatory regions, and decreased protein hormone receptor activity. Conversely, Tb^+^ was characterized by downregulation of biological processes related to epithelial transport and maturation, elastin turnover, and indole biosynthesis. Cellular component terms continued to involve receptor adhesion and trafficking modules, such as the integrin αvβ3 complex, clathrin-linked vesicles, the SNARE complex, and type III intermediate filaments, while molecular functions showed reductions in prostaglandin-D synthase, JUN kinase, NSF-adapter, and esterase activities. (Figure 11). Supplementary File S4 provides detailed information on the enriched pathways in HC along with the corresponding gene counts.

#### 2.7.5. KEGG Pathway Analysis of Head Cartilage (HC) DEGs

KEGG pathway analysis of HC revealed distinct enrichment profiles across the experimental groups (Supplementary File S5). In the Mc group, pathways enriched among upregulated genes included the JAK-STAT signaling pathway, PPAR signaling pathway, Th17 cell differentiation, phagosome, and neutrophil extracellular formation. The Mc^+^ group exhibited enrichment of pathways related to glycosphingolipid biosynthesis, glycerolipid metabolism, porphyrin metabolism, PPAR signaling pathway, and Fc gamma R-mediated phagocytosis. In the Tb group, pathways enriched among upregulated genes comprised the VEGF signaling pathway, PPAR signaling pathway, IL-17 signaling pathway, inflammatory mediator regulation of TRP channels, and the MAPK signaling pathway. The Tb^+^ group showed significant upregulation of pathways associated with phagosome, Fc gamma R-mediated phagocytosis, JAK-STAT signaling pathway, natural killer cell-mediated cytotoxicity, and leukocyte transendothelial migration.

**Figure 12:**
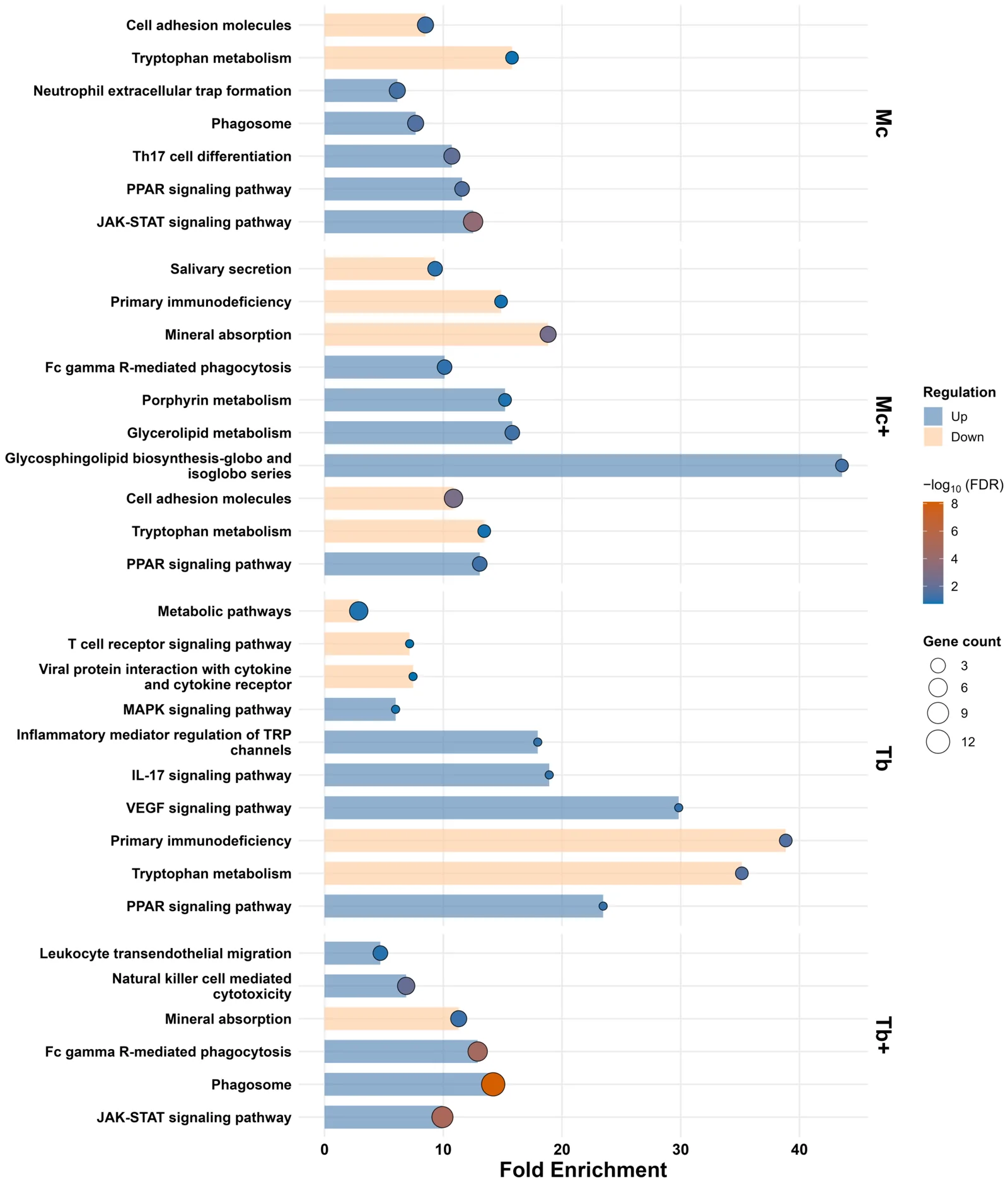
Significantly regulated KEGG pathways in infected head cartilage.

KEGG analysis in HC also showed pathways enriched among downregulated genes across the groups. In the Mc group, decreased expression was associated with cell adhesion molecules and tryptophan metabolism. The Mc^+^ group showed downregulation in pathways related to mineral absorption, primary immunodeficiency, tryptophan metabolism, and cell adhesion molecules. In the Tb group, pathways enriched among downregulated genes included primary immunodeficiency, viral protein interaction with cytokine and cytokine receptor signaling pathway, metabolic pathways, and Th17 cell differentiation. The Tb^+^ group exhibited reduced activity in the mineral absorption pathway (Figure 12). Supplementary File S7 shows the enriched KEGG pathways in HC, along with the number of identified genes.

These findings are based on RNA-seq analysis; therefore, immune cell-related terms should be interpreted as transcriptional signatures rather than direct evidence of changes in specific immune cell populations.

### 2.8. Validation of Potential KEGG Pathways in the Kidney and the Head Cartilage (HC)

**Figure 13:**
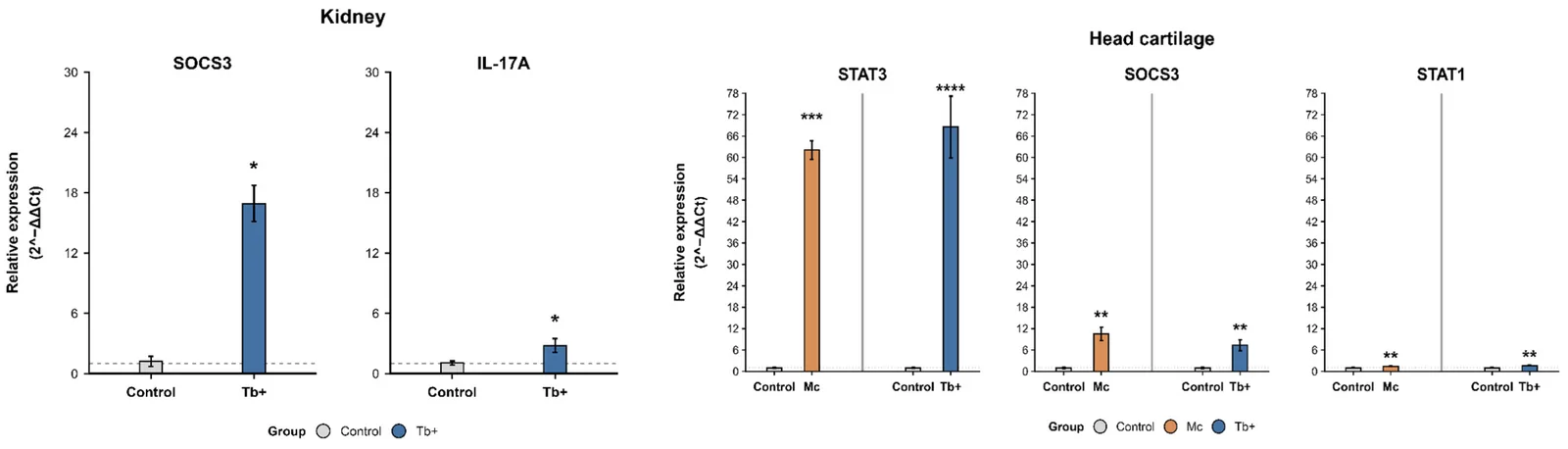
RT-qPCR validation of selected DEGs in the kidney and head cartilage. Asterisks indicate statistical significance: * *p* < 0.05, ** *p* < 0.01, *** *p* < 0.001, and **** *p* < 0.0001.

RT-qPCR analysis confirmed the potential KEGG pathways obtained by RNA-seq analysis (Figure 13), supporting the reliability of the transcriptomic findings in the kidney and head cartilage. In the Tb^+^ kidney, SOCS3 and IL-17A were significantly upregulated, while STAT1 showed upregulation but was not significant, collectively supporting coordinated regulation of JAK-STAT signaling and the IL-17-related immune response and confirming the indicated pathways. In the Mc kidney, IL17A was downregulated by both RNA-seq and RT-qPCR, although the RT-qPCR downregulation was not statistically significant. The simultaneous upregulation of SOCS3, STAT3, and STAT1 in the Mc and Tb^+^ group of HC was consistent with proposed activation of JAK-STAT signaling in these groups.

Relative expressions of SOCS3 and IL17A were measured in comparison to the control group in kidney (left) samples from the Tb^+^ group, while SOCS3, STAT1, and STAT3 were measured in HC (right) from the Mc and Tb^+^ groups.

## 3. Discussion

RNA-seq has been widely used in transcriptomic studies of myxozoan parasites [27], including investigations of immune responses in *T. bryosalmonae*-infected trout [25] and *M. bejeranoi*-infected tilapia [26], as well as comparative analyses in salmonids [28]. Recent studies have examined transcriptional responses during single and dual infections with *T. bryosalmonae* and *M. cerebralis* [29]. In this study, kidney and HC were sampled at 90 days post-infection (dpi) or 60 days post-co-infection (dpc) to assess organ responses during established disease, when parasite progression is evident [13]. DEG counts (Figure 2) and clustering patterns (Figure 5) indicate tissue tropism largely influences transcriptomic response magnitude, with stronger responses in the kidney during *T. bryosalmonae* and in cartilage during *M. cerebralis* infection. Infection sequence also impacts responses, as Tb^+^ shows increased transcriptional changes in cartilage following prior *T. bryosalmonae* infection and subsequent *M. cerebralis* exposure. *T. bryosalmonae* induces the largest transcriptomic response in the kidney, consistent with renal tropism, while in HC, Mc and Tb^+^ cause marked transcriptomic changes, reflecting cartilage as a major target for *M. cerebralis*.

### 3.1. Transcriptomic Response to M. cerebralis Single Infection (Mc)

NK cells are part of the innate immune system and mediate cytotoxicity in fish [30]. Genes annotated to NK cell-mediated cytotoxicity, including *nectin2* and *pvr*, were enriched in the kidneys of Mc fish. This pattern indicates modulation of cytotoxic activity. However, the analysis was performed on bulk kidney tissue, and it is difficult to ensure whether such modulation results from activation, increased abundance of NK-like cells, or non-specific cytotoxic cells (NCCs). Mast cells (MCs) can recruit and influence NK cells through IFN-dependent mediators. In fish, MCs are key innate immune cells, especially at mucosal sites. In myxozoan-infected *Chelon ramada*, mast-cell recruitment and degranulation near parasite spores correlate with inflammation, indicating roles in parasite detection and defense [31].

In salmonids, eosinophilic granule cells (EGCs), similar to MCs, are more abundant in disease-resistant species like coho salmon, highlighting their protective role. Infection in zebrafish with *Pseudocapillaria tomentosa* shows abundant EGCs in the peritoneum and kidney, linked to anti-parasitic responses. In teleosts, EGCs may accumulate at parasite sites, and increased recruitment and degranulation are linked to chronic inflammation. Our dataset shows enrichment in eosinophil pathways, primarily downregulated, suggesting a possible reduction in eosinophil activity during infection.

Macrophages in teleosts are vital to innate immunity, showing M1-like pro-inflammatory and M2-like anti-inflammatory states [32,33]. Enrichment of pathways regulating macrophage inflammatory protein 1-alpha suggests their role in inflammation during *M. cerebralis* infection in the kidney, supporting the “macrophage-first” model where early macrophage responses influence host–parasite interactions [32]. Enriched pathways linked to activated CD8^+^ αβ T cell apoptosis indicate CD8^+^ T cell involvement; in teleosts, these cells mediate cytotoxicity through perforin, proteases, and FasL pathways, regulating infected cells and T cell homeostasis during parasite exposure in gibel carp [34,35] and Nile tilapia [36].

Pathways related to CCR signaling, ECM organization, and ICAM-3 were mostly downregulated, possibly reflecting reduced immune cell recruitment and parasite immune evasion, indicating a systemic immune response during WD. In zebrafish, CCR expression has been observed across tissues and developmental stages [37], and in Atlantic salmon, their repertoire is expanded through whole-genome duplication, with several linked to inflammatory responses [38]. This pattern may reflect reduced chemokine-mediated immune cell recruitment, which could contribute to parasite persistence and immune evasion [39].

HC showed a stronger transcriptomic response, including enrichment of Th17 and IL-23 pathways. Th17 cells, crucial against extracellular pathogens, differentiate via STAT3 [40], consistent with activation of a pro-inflammatory response to the *M. cerebralis* infection [41]. However, the analysis indicates a Th17-associated transcriptional signature rather than direct evidence of Th17 cell differentiation.

Upregulation of phagolysosome genes suggests increased transcription linked to phagolysosomal activity, consistent with responses during *Aeromonas hydrophila* infection in *Oreochromis niloticus* [42]. Cytokine signaling pathways (*cntf*, *lif*, and *osm*) involved in tissue repair mechanisms and neuro-immune regulation may highlight roles in hematopoiesis, tissue regeneration, and neuronal repair [43,44]. These findings are solely based on transcriptional evidence.

Conversely, pathways associated with oligodendrocyte progenitor and neuronal projection were downregulated, possibly implying impaired axonal growth and connectivity [45], which may be due to inflammation or parasite damage [46]. Evidence of neuronal damage and disrupted spinal coordination has been observed histologically during WD [47]; however, the present findings are based upon transcriptomic evidence.

### 3.2. Transcriptomic Response to Co-Infection with T. bryosalmonae (Mc^+^)

In the Mc^+^ kidney, enriched BP terms such as immune complex clearance, plasma cell differentiation, and cytokine signaling suggest coordinated activation of an innate and adaptive immunity-associated transcriptional signature. Similar immune responses were found in rainbow trout gills and fins infected with *M. cerebralis*, involving cytokine and antigen-presentation pathways [29].

Enrichment of eosinophil chemotaxis further suggests transcriptional modulation of inflammatory and possible tissue remodeling processes, as seen in co-infections with Lepeophtheirus *salmonis* and ISAv in *Salmo salar*, which activates IFN signaling, Toll-like receptor, and MHC-I expression [29,48]. Enrichment of terms associated with endocytic vesicles, lipoproteins, and immunological synapse points to antigen processing transcriptional activity consistent with vesicle-mediated transport during co-infection studies [10]. Molecular functions such as IgG receptor, CX3C, CCR3, and MHC II binding indicate a strong humoral and T-cell-associated transcriptional signature, aligning with other co-infection models [49]. Co-infections can also modulate immunity, where increased signaling may be paired with parasite-mediated interference in effective parasite clearance [48].

Suppression of immune and metabolism-related pathways suggests transcriptional modulation of host oxidative and metabolic stress responses. In co-infected Atlantic salmon, immune activation coincided with downregulation of metabolic and antigen processing genes [49]. Key cytokine pathways such as *IL-23*, *Th17*, *IL-23R*, *IL-12R*, and *IL-6R* were also downregulated, contrasting with a single-infection study of *M. cerebralis*, where Th17 signaling was upregulated, suggesting co-infection alters cytokine dynamics [48]. This may reflect SOCS-mediated feedback suppression, a known immune-evasion strategy of myxozoans [14].

In HC, the Mc^+^ group showed upregulation of genes associated with metabolic processes, ECM, and immune-related pathways. This transcriptional pattern is consistent with macrophage activation and tissue repair [48]. Phagolysosome enrichment further indicates increased transcription related to macrophage-mediated degradation, a key antimicrobial mechanism involving lysosomal enzymes, nitric oxide, and ROS production [42].

Genes related to epithelial integrity, kidney development, and lymphocyte differentiation were downregulated, suggesting impaired tissue homeostasis and immune-development-related transcription. Similar immunosuppression occurs in co-infected Atlantic salmon, where sea lice and ISAv reduce the expression of genes involved in T-cell activation, ECM organization, and cytoskeletal maintenance [36,50]. Suppression of cytoskeletal and vesicle-trafficking components may further impair transport and immune function [33,34]. Reduced ammonium transporters and ion symporters suggest altered osmoregulation, as reported during systemic infections in fish [49]. These transcriptional changes may indicate impaired tissue homeostasis and immune competence, supporting parasite persistence.

Overall, the comparison between single and sequential infections indicates that co-infection modifies transcriptomic responses rather than simply amplifying host responses. In the kidney, the transition from Mc to Mc^+^ was associated with intensified immune signaling, along with selective suppression of cytokine and antigen-presentation pathways, suggesting complex host–parasite interactions during co-infection. In HC, a slight decrease in DEGs during co-infection suggests a more focused transcriptional response while maintaining inflammatory and macrophage-mediated defense pathways.

### 3.3. Transcriptomic Response to T. bryosalmonae Single Infection (Tb)

In *T. bryosalmonae*-infected kidneys, enrichment of BP, including chronic inflammation, immune complex clearance, and *IL-8* responses, indicates a strong proinflammatory-related transcriptional response. Similar patterns have been reported in PKD studies, showing increased B and T cell markers, hypergammaglobulinemia, and macrophage activation in trout kidneys [14,51]. The enrichment of T-cell receptor, IgG receptor, chemokine receptor, and IL-1/IL-2 pathways aligns with previous proteomic findings, which identified regulation of MHC II, immunoglobulins, complement C3, and immune proteins in the kidney, supporting innate and adaptive immune activation [51,52].

Kidney transcriptomics revealed downregulation of genes associated with xenobiotic glucuronidation, flavonoid metabolism, leukotriene catabolism, and MHC Ib-mediated lipid antigen presentation. It suggests transcriptional suppression of metabolic and immune-related processes during chronic infection, which may contribute to reduced inflammation and parasite persistence [9,51]. Differential regulation of leukotriene-B4 20-monooxygenase and phenanthrene monooxygenase may further modulate immune-related pathways [53]. Reduced IL-23 receptor signaling and MHC-mediated antigen presentation align with previous proteomic data showing differential abundance of MHC II, complement C3, immunoglobulins, and immune proteins in the kidney [52]. Collectively, these findings suggest coordinated transcriptional modulation of antigen presentation and immune response pathways during *T. bryosalmonae* infection.

In HC, *T. bryosalmonae* triggered pathways related to lipid metabolism, corticosteroid secretion, and lysosomal enzymes. Although cartilage is not the primary target organ for *T. bryosalmonae*, these responses suggest a local metabolic response [54,55]. Regulation of aldosterone and mineralocorticoid secretion may further suggest endocrine-immune interaction, aligning with mineralocorticoids’ role in macrophage regulation in teleosts [56,57]. Elevated metalloaminopeptidase-associated activity likely reflects transcriptional changes related to tissue remodeling, as reported during *Sphaerospora molnari* infection [58].

Suppression of pathways associated with B-cell development, thyroid hormone signaling, and antigen presentation suggests suppression of immune-related transcription. Downregulation of genes involved in B-cell maturation, such as *cd79a*, *cd22*, and *bcl7*, suggests disruption of transcriptional processes linked to B-cell development during PKD [59]. Reduced TSH pathway activity aligns with thyroid hormones’ role in lymphocyte and macrophage function in rainbow trout [60], potentially disturbing immune balance. Decreased MHC class I binding and T cell components imply impaired antigen processing and T-cell activation, supporting PKD immune evasion [51]. Together, these changes, along with reduced ferroxidase and prostaglandin-D synthase activity, may weaken inflammatory and oxidative responses at the transcription level.

### 3.4. Transcriptomic Response to Co-Infection with M. cerebralis (Tb^+^)

In Tb^+^ kidneys, biological processes like immune complex clearance, MIF signaling, and IL-8 response indicate a pronounced pro-inflammatory transcriptional response involving pathways related to myeloid cell recruitment activity. The MIF-CD74 complex supports macrophage survival and cytokine regulation in parasitic infections like PKD [48,53]. Enrichment of genes related to vesicles, ER chaperones, eosinophils, and chemokine receptors suggests enhanced phagocytosis and leukocyte recruitment [61,62]. Upregulation of *cxcr2* and mast-cell pathways suggests enhanced mast-cell activity during co-infection, consistent with mast-cell-macrophage responses reported in teleost infections [61]. Enrichment of T-cell receptor and IL-2 receptor-associated terms implies T-cell-related transcriptional signaling, whereas mast-cell granule and chemokine receptor-associated terms show a coordinated immune cell recruitment pathway. Elevated plasma-cell differentiation, IgG receptor activity, and antigen presentation suggest strong transcription of B-cell responses. Downregulation of IL-23, Th17, TLR8, and IL-12/IL-23 receptor suggests impaired pathogen recognition, reduced cytokine responsiveness, and weakened innate immunity-related pathways, aligning with reports of diminished Th17 responses in PKD [14,63].

Suppression of IL-23 and IL-12 receptors may reduce cytokine responsiveness, and metabolic suppression hints at altered host metabolism and redox regulation, as seen in other myxozoan infections. Overall, co-infection suppresses cytokine signaling, antigen presentation, and innate immunity, consistent with known weak Th1 and Th17 responses during late PKD [53,64].

In HC, upregulation of IL-23-, Th17-, IL-12-, and IL-6 pathways, may indicate activation of pro-inflammatory, Th1, and Th17 related transcriptional responses [65,66,67]. Increased B-cell differentiation, IL-3 signaling, Fc-gamma and IgG receptor activity may suggest enhanced humoral, hematopoietic, and antibody-mediated immune functions at the transcriptional level [53,59,68]. Upregulation of the arachidonate 8(S)-lipoxygenase pathway and *CNTFR* complex indicates inflammatory regulation and neuroimmune interaction-related transcriptional activation during chronic infection [44,69].

The downregulation of developmental and immune processes in HC suggests a reduction in the gene expression profile related to immunoregulation and regeneration during co-infection. Reduced elastin catabolism and indole biosynthesis provide transcriptomic evidence of compromised ECM remodeling and tryptophan-based defense [14,70].

In the kidney, co-infection following Tb exposure seems to reshape the transcriptional response from strong inflammation and metabolic activation toward a complex but modulated immune reaction, involving macrophages, eosinophils, and T cells, while suppressing major cytokines. In HC, transition from Tb to Tb^+^ indicates an increased transcriptional response and shifts the response from predominant inflammation toward remodeling and endocrine-modulated immune regulation, accompanied by suppression of adaptive immune pathways. A comparative summary of the major immune and metabolic pathways enriched in the kidney and head cartilage across all infection groups is presented in Supplementary File S8.

### 3.5. KEGG Pathway Analysis at the Target Sites

Both the Mc^+^ and *T. bryosalmonae*-infected groups showed enrichment in cytokine receptors, chemokines, T-cell receptors, along with enhanced antigen presentation, and Th1, Th2, and Th17 signaling pathways indicating transcriptomic coordination of innate and adaptive immune responses [48,64]. These are consistent with previous reports of increased IgT^+^ B-cell abundance and elevated antigen-presenting molecules during myxozoan infections [13]. The Tb^+^ group exhibited enrichment of IL-17, JAK-STAT, TNF, T cell receptor, and cytokine-cytokine receptor signaling, reflecting a heightened inflammatory state, aligning with activation of macrophage, eosinophil, and T cell-related pathways in co-infected kidneys [14,62], and is reinforced by increased expression of *stat1*, *stat3*, and components of the MIF-CD74 axis [59].

Downregulated pathways in the Mc group included IL-17 signaling, chemokine signaling, cytokine receptor interaction, HIF-1 signaling, and amino acid biosynthesis, directing immune suppression and a reduced inflammatory transcription pattern [14]. In the Mc^+^ group, downregulation of JAK-STAT signaling, phagosome, and amino acid biosynthesis suggests weakened immune signaling and metabolic conservation, consistent with SOCS3 inhibiting *STAT1* and *STAT3* [14,64]. In *T. bryosalmonae*-infected trout, suppressed PPAR signaling, steroid biosynthesis, xenobiotic metabolism, and fructose-mannose pathways may indicate metabolic reprogramming during chronic PKD [65,71]. The Tb^+^ group showed downregulation of steroid hormone biosynthesis, hematopoietic cell lineage, AMPK signaling, nitrogen metabolism, and mineral absorption, possibly reflecting disrupted energy metabolism and impaired cellular development under chronic immune stress [66,71].

In the HC, both single and co-infection groups exhibited upregulation of immune and metabolic pathways. Phagosome and Fc gamma R-mediated phagocytosis were enriched in the Mc^+^ and Tb^+^ groups, suggesting heightened innate immune activity and macrophage responses related to transcriptional expression, consistent with earlier fish-parasite studies [53] and co-infection conditions [10]. The JAK-STAT and Th17 pathways, upregulated in the Mc, Tb, and Tb^+^ groups, suggest transcriptional activation of T cell-driven adaptive responses against *T. bryosalmonae* and *M. cerebralis*, aligning with previous data in resistant trout [9,48]. JAK-STAT promotes T helper cell polarization in teleost mucosa. PPAR signaling, enriched in the Mc and Tb groups, suggests a link between lipid metabolism and immune regulation, inflammation, and tissue remodeling [69]. Glycerolipid metabolism and glycosphingolipid biosynthesis in the Mc^+^ group suggest metabolic activity in skeletal tissue during chronic infection, related to membrane turnover, ECM remodeling, and oxidative stress [69].

Suppression of cell adhesion molecules (CAMs) and tryptophan metabolism in Mc and Mc^+^ groups may indicate parasite-induced disruption of cell–cell communication and amino acid homeostasis. Tryptophan derivatives (e.g., kynurenines) are critical immunomodulators in fish, and their dysregulation may facilitate immune evasion [13]. In the Tb group, downregulation of Th17 differentiation, primary immunodeficiency, and T cell receptor signaling suggests a potential suppression of adaptive T-cell responses and antigen presentation. This agrees with findings showing that *T. bryosalmonae* impairs CD8^+^ T cell responses and MHC expression in susceptible hosts [9,14]. Moreover, the upregulation of the expression of SOCS3 and IL-17A in the Tb^+^ kidney suggests activation of the Th17-associated pathway and the SOCS3-mediated negative feedback on STAT3. Similar increases in IL-17A and SOCS3 have been associated with dysregulated Th17 response in *M. cerebralis*-infected susceptible rainbow trout, whereas increased STAT3 activity was linked to controlled immune response [72]. While the decrease in IL-17A in Mc and the increase in Tb^+^ might suggest that *M. cerebralis* suppresses the fish immune response, which could be a part of the parasite evasion strategy to establish the infection and promote its growth. In the head cartilage, the concurrent upregulation of STAT1, STAT3, and SOCS3 in the Mc and Tb^+^ groups suggests activation of JAK-STAT signaling accompanied by SOCS3-mediated negative feedback regulation. STAT3 is involved in Th17 differentiation, whereas SOCS3 can restrict IL-23 signaling; therefore, this expression pattern may reflect active but tightly regulated local immune signaling within the head cartilage [72]. Immune and metabolic pathways enriched in the kidney and head cartilage of rainbow trout during single and co-infection with *M. cerebralis* and *T. bryosalmonae* are summarized in Supplementary File S8.

The analysis used human orthologs for functional annotation [73,74,75] because they provide broader functions and pathways than available rainbow trout annotations. While this approach improves the detection of conserved immune signaling and metabolic processes, it may favor pathways more extensively studied in humans and might potentially miss teleost-specific gene functions or regulatory interactions. Therefore, these pathways are regarded as markers of conserved functions rather than definitive evidence that the complete human pathway exists in rainbow trout.

## 4. Integration of Transcriptomic and Previous Proteomic Findings

The present transcriptomic analysis complements a recent quantitative proteomic study, which examined the same infection model, target organs (kidney and head cartilage), and infection groups [52]. Both approaches identified activation of immune-related pathways, including cytokine signaling, JAK-STAT signaling, IFI44, antigen presentation, macrophage activation, complement responses, and oxidative stress. Despite these similarities, RNA sequencing captured a substantially broader molecular landscape, identifying 47,152 expressed genes, whereas the proteomic study detected comparatively fewer differentially abundant proteins. This difference reflects the greater sensitivity and dynamic range of RNA sequencing, while proteomic analyses are inherently constrained by protein abundance, extraction efficiency, peptide detectability, and post-transcriptional regulation. Consequently, many transcriptional changes are not expected to directly correlate with protein abundance.

## 5. Materials and Methods

### 5.1. Study Design and Sample Collection

**Figure 14:**
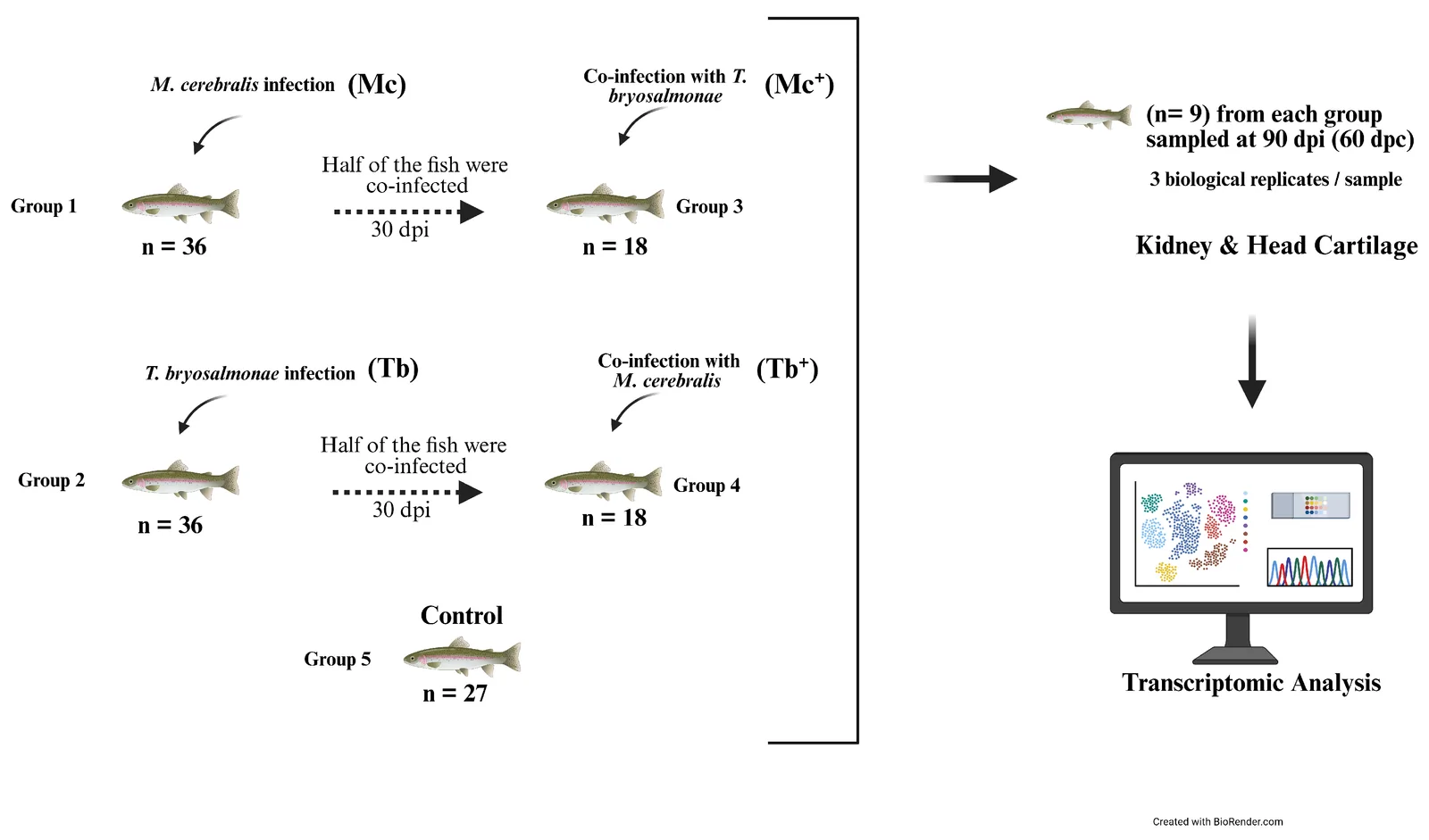
Illustration of the in vivo experimental design showing the infection setup, experimental groups, and sampling points.

Experimental infections were performed as previously described in our earlier study [29]. Briefly, pathogen-free North American Strain Trout Lodge (TL) rainbow trout (3 months old, mean length 4.02 ± 0.26 cm, mean weight 0.6 ± 0.15 g) were obtained from Forellenzucht Trostadt GmbH & Co. KG (Reureith, Germany) and were exposed to *M. cerebralis* and *T. bryosalmonae*. Thirty days post-infection (dpi), half of the fish from each infected group were reciprocally challenged with the other parasite, separately (Figure 13). At 90 dpi, fish from each group were euthanized in buffered tricaine methanesulfonate (MS-222) overdose (250–500 mg/L) [76]. Death was confirmed before the collection of Kidney and Head cartilage samples, which were stored at −80 °C until further analysis. During acclimatization and throughout the trial, fish were monitored for mortality, abnormal behavior, external lesions, and other clinical signs of disease. Additionally, using BF-2 and EPC cell lines, homogenates of the brain, whole viscera, spleen, and kidney were subjected to viral screens before and during the experiment in accordance with conventional cell culture procedures [77]. No virus was identified at any point before or during the experiment. Water quality parameters were regularly monitored and maintained within the recommended ranges to minimize environmental stress and potential contamination-related effects. The experimental design and sampling strategy are illustrated in Figure 14.

### 5.2. RNA Extraction and Sequencing

Total RNA was isolated from the kidney and head cartilage (HC) collected at 90 dpi. For each tissue, samples from 15 fish were processed (3 fish per group; 5 experimental groups in total), using a RNeasy Mini Kit (Qiagen, Hilden, Germany) following the manufacturer’s protocol. An on-column DNase digestion step was included to remove residual genomic DNA. RNA quantity and purity were assessed with a NanoDrop 2000c spectrophotometer (Thermo Fisher Scientific, Wilmington, NC, USA), and integrity was checked by agarose gel electrophoresis. Only samples with RNA integrity numbers (RIN) ≥ 8.0 were used for library preparation.

For each sample, 500 ng of total RNA were subjected to mRNA enrichment using the Lexogen Poly(A) RNA Selection Kit V1.5 (Lexogen, Vienna, Austria). mRNA-enriched fractions were then used to generate cDNA libraries with the CORALL mRNA-Seq Library Prep Kit (Lexogen), according to the manufacturer’s instructions, yielding a total of 30 libraries (15 kidney and 15 HC). Library size distribution and quality were evaluated using a High Sensitivity D1000 ScreenTape Kit (Agilent Technologies, Santa Clara, CA, USA) on the Agilent 4200 TapeStation System. Sequencing was performed at the NGS unit of the Vienna BioCenter Core Facilities (VBCF, Vienna, Austria) on an Illumina NovaSeq 6000 platform, generating 150 bp paired-end reads. The RNA-seq datasets analyzed during the current study are available in the ArrayExpress repository under accession number E-MTAB-16908.

### 5.3. Quantitative RT-PCR

For infection confirmation and assessing the parasite load of *M. cerebralis* and *T. bryosalmonae* from kidney and HC, we used Myx18 (Myx18-909F/996R) and RPL18 (RPL18F/R) as described previously [78,79]. Reaction 20 µL contained 4 µL of 1:10 diluted cDNA, 1× SsoAdvanced Universal SYBR Green Supermix (Bio-Rad, Munich, Germany), 0.4 µM of each primer, and nuclease-free DEPC-treated water. *lif* Amplification was performed on a CFX96 Touch Real-Time PCR system (Bio-Rad) with initial denaturation at 95 °C for 5 min, followed by 35 cycles of 95 °C for 30 s, 57–62 °C for 30 s, and 72 °C for 30 s. Amplicon specificity was confirmed by melting curve analysis (55–95 °C). Elongation factor 1-alpha (*EF1α*) was used as the reference gene for normalization [80]. Detection thresholds for Myx18 and RPL18 were 36.43 and 37.61, respectively [48]. Relative expression levels were calculated using CFX Manager software v3.1 (Bio-Rad) (Figure 1).

### 5.4. Read Mapping and Expression Profiling of Host Genes

To avoid interference with downstream analysis, the Trimmomatic v0.39 (Galaxy implementation) was used to remove adapter sequences [81]. The sequence data were analyzed using CLC Genomics Workbench 23.0.5 (Qiagen, Aarhus, Denmark). The raw sequence reads were subjected to quality control by using parameters such as Phred score, read length, and adapter sequences [28]. Quality filtering was performed with an error probability threshold of 0.01, allowing a maximum of two ambiguous nucleotides per read. Bases with a Phred quality score ≤ 30 were removed, and reads shorter than 50 nucleotides or longer than 150 nucleotides, as well as adapter and unique molecular identifier (UMI) sequences from library preparation, were discarded. Genome data for rainbow trout were obtained from NCBI and used as a reference. Trimmed reads were mapped against the rainbow trout genome reference (USDA_OmykA_1.1, GCF_013265735.2) using the default mapping parameters of the CLC Genomics RNA-seq tool (mismatch cost = 2, insertion cost = 3, deletion cost = 3, length fraction = 0.8, similarity fraction = 0.8). For differential expression, infected groups (Mc, Mc^+^, Tb, and Tb^+^) were compared pairwise to the respective non-infected control tissues. Genes exhibiting a log_2_ fold change of >1 and a false discovery rate (FDR)-adjusted *p*-value ≤ 0.01 were considered differentially expressed. Ortholog mapping for cross-species gene annotation was performed using g: Profiler [82]. Compared to rainbow trout, human genes have a broader availability of annotation resources and biological knowledge. So, rainbow trout gene symbols were converted to human orthologs [73]. Zebrafish orthologs were also identified; however, Gene Ontology (GO) term coverage for zebrafish was limited in comparison to human annotation. GO categories cellular component (CC), molecular function (MF), and biological process (BP), as well as Kyoto Encyclopedia of Genes and Genomes (KEGG) pathways enriched among annotated DEGs, were analyzed using an FDR cut-off of 0.05 in ShinyGO 0.80 [83].

### 5.5. Validation of Potential KEGG Pathways by qRT-PCR

To validate the potential KEGG pathways identified by the RNA-seq, we performed quantitative real-time PCR (qRT-PCR) on immune signaling-related differentially expressed genes. The analyzed genes included STAT3, STAT1, SOCS3, and IL17A. The primer sequences used for these genes were adopted from previously published studies (Supplementary File S9). RNA extracted from samples was utilized to synthesize cDNA using the iScript cDNA Synthesis Kit (Bio-Rad, Munich, Germany). Three independent biological replicates per group were subjected to qRT-PCR. The reaction was performed in a final volume of 10 µL, containing 3 µL (100 ng/µL) of 1:10-fold-diluted cDNA samples, 0.5 µM of each primer, 1× SsoAdvanced™ Universal SYBR Green Supermix (Bio-Rad), and DEPC-treated sterile distilled water. The specificity of each assay was confirmed by melt-curve analysis, which produced a single amplification peak. Three independent biological replicates were analyzed per experimental group, and each biological replicate was measured in technical duplicate. *EF1-α* was used as a reference gene for normalization [84]. The mean Cq value of the technical replicates was used for calculations. The 2^−ΔΔCt^ calculation method was used to determine the relative gene expression of the DGEs. The statistical difference between the control and infected groups was determined using an unpaired *t*-test with Welch’s correction. For statistical tests, a *p*-value of < 0.05 was regarded as significant. Consistency between the RNA-seq and RT-qPCR results was evaluated by comparing the direction of gene expression changes observed by the two methods.

### 5.6. Comparative Analysis with Proteomic Study

We evaluated the consistency and integration of our transcriptomic findings by comparing the differentially expressed genes (DEGs) and enriched pathways with those identified in a recent proteomic study on *T. bryosalmonae* and *M. cerebralis* infections in rainbow trout [52]. By cross-referencing key immune pathways, genes, and tissue-specific responses, we aimed to uncover both shared and distinct host responses in the kidneys and head cartilage.

### 5.7. Statistical Approach

Differential gene expression was assessed in CLC Genomics Workbench (Qiagen) using the RNA-Seq differential expression module, which applies a negative binomial generalized linear model (multi-factorial design) to read counts and applies TMM (trimmed mean of M-values) normalization.

## 6. Conclusions

Host transcriptional responses to myxozoan infection differed between single and sequential co-infections and appeared to be influenced by tissue tropism. The transcriptomic patterns observed here suggest that infection sequence is associated with tissue-dependent changes in host transcriptional profiles, rather than a uniform increase or decrease in response intensity. In the kidney, the transition from Mc to Mc^+^ was associated with an increased number of DEGs. In contrast, the shift from Tb to Tb^+^ led to fewer DEGs, suggesting that prior kidney infection with *T. bryosalmonae* may modulate the transcriptional response to subsequent *M. cerebralis* exposure. In HC, the opposite pattern was observed: the transition from Mc to Mc^+^ was associated with a reduced transcriptional response, whereas the shift from Tb to Tb^+^ increased DEG numbers. These contrasting patterns are consistent with the distinct tissue preferences of the two parasites, with *T. bryosalmonae* primarily targeting the kidney and *M. cerebralis* targeting cartilage tissues. Overall, these findings suggest that sequential co-infection results in tissue-specific modulation of host transcriptional responses dependent on parasite tropism and infection order. This study presents transcriptional signatures using a late infection time point, which limits the interpretation of dynamic immune processes. However, it enhances the understanding of sequential *T. bryosalmonae* and *M. cerebralis* infections and provides a basis for identifying tissue-specific biomarkers and key immune targets. The findings may support the development of improved monitoring methods and control of myxozoan infections in salmonids. Future studies investigating changes in specific immune cell populations, immunohistochemistry, or in situ hybridization would further clarify how tissue tropism and infection sequence shape host responses during myxozoan co-infection.

## Data Availability

The original contributions presented in this study are included in the article/Supplementary Materials. The RNA-seq datasets analyzed during the current study are available in the Array Express repository under accession number E-MTAB-16908. Further inquiries can be directed to the corresponding author.

## Supplementary Materials

The following supporting information can be downloaded at: https://www.mdpi.com/article/10.3390/ijms27167253/s1. References [79,85,86] are cited in the supplementary materials.

## Abbreviations

PKD	Proliferative Kidney Disease
WD	Whirling Disease
HC	Head Cartilage
SOCS	Suppressor of Cytokine Signaling
STAT	Signal Transducer and Activator of Transcription
*CNTF*	Ciliary Neurotrophic Factor Receptor Activity
GO	Gene Ontology
KEGG	Kyoto Encyclopedia of Genes and Genomes
TNF	Tumor Necrosis Factor
IFI44	Interferon-Induced Protein 44
RSAD2	Radical S-adenosyl Methionine Domain Containing 2
ISG15	ISG15 Ubiquitin-Like Modifier

## References

[1] Buchmann K. (2002). Interactions between Monogenean Parasites and Their Fish Hosts. Int. J. Parasitol..

[2] Knudsen R., Amundsen P.-A., Jobling M., Klemetsen A. (2008). Differences in Pyloric Caeca Morphology between Arctic Charr *Salvelinus Alpinus* Ecotypes: Adaptation to Trophic Specialization or Parasite-induced Phenotypic Modifications?. J. Fish Biol..

[3] Al-Jahdali M., Hassanine R. (2010). Ovarian Abnormality in a Pathological Case Caused by Myxidium Sp. (Myxozoa, Myxosporea) in Onespot Snapper Fish Lutjanus Monostigma (Teleostei, Lutjanidae) from the Red Sea. Acta Parasitol..

[4] Canning E.U., Tops S., Curry A., Wood T.S., Okamura B. (2002). Ecology, Development and Pathogenicity of Buddenbrockia Plumatellae Schröder, 1910 (Myxozoa, Malacosporea) (Syn. Tetracapsula Bryozoides) and Establishment of Tetracapsuloides n. Gen. for Tetracapsula Bryosalmonae. J. Eukaryot. Microbiol..

[5] Canning E.U., Okamura B. (2004). Biodiversity and evolution of the Myxozoa. Adv. Parasitol..

[6] El-Matbouli M., Mattes M., Soliman H. (2009). Susceptibility of Whirling Disease (WD) Resistance and WD Susceptible Strains of Rainbow Trout *Oncorhynchus mykiss* to *Tetracapsuloides bryosalmonae*, *Yersinia ruckeri* and Viral Haemorrhagic Septicaemia Virus. Aquaculture.

[7] Sarker S., Kallert D.M., Hedrick R.P., El-Matbouli M. (2015). Whirling Disease Revisited: Pathogenesis, Parasite Biology and Disease Intervention. Dis. Aquat. Org..

[8] Oumouna M., Hoffmann R.W. (2011). Susceptibility of Rainbow Trout (*Oncorhynchus mykiss*) and Carp (*Cyprinus carpio*) to *Myxobolus cerebralis*, the Agent of Whirling Disease. J. Cell Anim. Biol..

[9] Sudhagar A., Kumar G., El-Matbouli M. (2019). The Malacosporean Myxozoan Parasite *Tetracapsuloides bryosalmonae*: A Threat to Wild Salmonids. Pathogens.

[10] Gorgoglione B., Bailey C., Bass D., Saraiva M., Adamek M., Noguera P., Ciulli S., Palíková M., Aguirre-Gil I., Bigarré L. (2020). Co-Infections and Multiple Stressors in Fish. Bull. Eur. Assoc. Fish Pathol..

[11] Kotob M.H., Menanteau-Ledouble S., Kumar G., Abdelzaher M., El-Matbouli M. (2017). The Impact of Co-Infections on Fish: A Review. Vet. Res..

[12] Klemme I., Louhi K., Karvonen A. (2016). Host Infection History Modifies Co-infection Success of Multiple Parasite Genotypes. J. Anim. Ecol..

[13] Kotob M.H., Gorgoglione B., Kumar G., Abdelzaher M., Saleh M., El-Matbouli M. (2017). The Impact of *Tetracapsuloides bryosalmonae* and *Myxobolus cerebralis* Co-Infections on Pathology in Rainbow Trout. Parasites Vectors.

[14] Kotob M.H., Kumar G., Saleh M., Gorgoglione B., Abdelzaher M., El-Matbouli M. (2018). Differential Modulation of Host Immune Genes in the Kidney and Cranium of the Rainbow Trout (*Oncorhynchus mykiss*) in Response to *Tetracapsuloides bryosalmonae* and *Myxobolus cerebralis* Co-Infections. Parasites Vectors.

[15] Read A.F., Taylor L.H. (2001). The Ecology of Genetically Diverse Infections. Science.

[16] Okon E.M., Okocha R.C., Taiwo A.B., Michael F.B., Bolanle A.M. (2023). Dynamics of Co-Infection in Fish: A Review of Pathogen-Host Interaction and Clinical Outcome. Fish Shellfish Immunol. Rep..

[17] Alarcón M., Thoen E., Poppe T.T., Bornø G., Mohammad S.N., Hansen H. (2016). Co-infection of *Nucleospora cyclopteri* (Microsporidia) and *Kudoa islandica* (Myxozoa) in Farmed Lumpfish, *Cyclopterus lumpus* L., in Norway: A Case Report. J. Fish Dis..

[18] Schmidt-Posthaus H., Steiner P., Müller B., Casanova-Nakayama A. (2013). Complex Interaction between Proliferative Kidney Disease, Water Temperature and Concurrent Nematode Infection in Brown Trout. Dis. Aquat. Org..

[19] Wang Z., Gerstein M., Snyder M. (2009). RNA-Seq: A Revolutionary Tool for Transcriptomics. Nat. Rev. Genet..

[20] Qian X., Ba Y., Zhuang Q., Zhong G. (2014). RNA-Seq Technology and Its Application in Fish Transcriptomics. OMICS.

[21] Salinas I., Magadán S. (2017). Omics in Fish Mucosal Immunity. Dev. Comp. Immunol..

[22] Sutherland B.J., Koczka K.W., Yasuike M., Jantzen S.G., Yazawa R., Koop B.F., Jones S.R. (2014). Comparative Transcriptomics of Atlantic *Salmo salar*, Chum *Oncorhynchus keta* and Pink Salmon *O. gorbuscha* during Infections with Salmon Lice *Lepeophtheirus salmonis*. BMC Genom..

[23] Yang Y., Yu H., Li H., Wang A. (2016). Transcriptome Profiling of Grass Carp (*Ctenopharyngodon idellus*) Infected with *Aeromonas hydrophila*. Fish Shellfish Immunol..

[24] Zhou R., Sun K., Xie X., Yin F., Galindo-Villegas J. (2025). Integrated Transcriptomic and Immune Enzymatic Analyses Uncover Coordinated Immunometabolic Responses in Large Yellow Croaker (*Larimichthys crocea*) to *Metanophrys* Sp. Infection. Front. Immunol..

[25] Ye H., Lin Q., Luo H. (2018). Applications of Transcriptomics and Proteomics in Understanding Fish Immunity. Fish Shellfish Immunol..

[26] Longshaw M., Le Deuff R., Harris A.F., Feist S.W. (2002). Development of Proliferative Kidney Disease in Rainbow Trout, *Oncorhynchus mykiss* (Walbaum), Following Short-term Exposure to *Tetracapsula bryosalmonae* Infected Bryozoans. J. Fish Dis..

[27] Shivam S., El-Matbouli M., Kumar G. (2021). Development of Fish Parasite Vaccines in the OMICs Era: Progress and Opportunities. Vaccines.

[28] Shivam S., Ertl R., Sexl V., El-Matbouli M., Kumar G. (2023). Differentially Expressed Transcripts of *Tetracapsuloides bryosalmonae* (Cnidaria) between Carrier and Dead-End Hosts Involved in Key Biological Processes: Novel Insights from a Coupled Approach of FACS and RNA Sequencing. Vet. Res..

[29] Akram N., Ertl R., Ghanei-Motlagh R., Secombes C.J., El-Matbouli M., Holzer A.S., Saleh M. (2025). Transcriptomic Analysis of the Rainbow Trout Response to Single and Co-Infections with *Myxobolus cerebralis* and *Tetracapsuloides bryosalmonae* at Sites of Parasite Entry. Int. J. Mol. Sci..

[30] Jørgensen J.B. (2014). The Innate Immune Response in Fish. Fish Vaccination.

[31] Dezfuli B., Castaldelli G., Tomaini R., Manera M., DePasquale J., Bosi G. (2020). Challenge for Macrophages and Mast Cells of Chelon Ramada to Counter an Intestinal Microparasite, *Myxobolus mugchelo* (Myxozoa). Dis. Aquat. Org..

[32] Wiegertjes G.F., Wentzel A.S., Spaink H.P., Elks P.M., Fink I.R. (2016). Polarization of Immune Responses in Fish: The ‘Macrophages First’ Point of View. Mol. Immunol..

[33] Hodgkinson J., Grayfer L., Belosevic M. (2015). Biology of Bony Fish Macrophages. Biology.

[34] Nakanishi T., Shibasaki Y., Matsuura Y. (2015). T Cells in Fish. Biology.

[35] Sukeda M., Shiota K., Kondo M., Nagasawa T., Nakao M., Somamoto T. (2021). Innate Cell-Mediated Cytotoxicity of CD8+ T Cells against the Protozoan Parasite *Ichthyophthirius multifiliis* in the Ginbuna Crucian Carp, *Carassius auratus langsdorfii*. Dev. Comp. Immunol..

[36] Li K., Zhu Y., Fang Z., Geng M., Zhang J., Zheng Y., Cao Y., Wei X., Yang J. (2025). Fish Requires FasL to Facilitate CD8+ T-Cell Function and Antimicrobial Immunity. J. Immunol..

[37] Liu Y., Chang M.X., Wu S.G., Nie P. (2009). Characterization of C–C Chemokine Receptor Subfamily in Teleost Fish. Mol. Immunol..

[38] Grimholt U., Hauge H., Hauge A.G., Leong J., Koop B.F. (2015). Chemokine Receptors in Atlantic Salmon. Dev. Comp. Immunol..

[39] Wiśniewska M.M., Kyslík J., Alama-Bermejo G., Lövy A., Kolísko M., Holzer A.S., Kosakyan A. (2025). Comparative Transcriptomics Reveal Stage-Dependent Parasitic Adaptations in the Myxozoan Sphaerospora Molnari. BMC Genom..

[40] Akram N., El-Matbouli M., Saleh M. (2023). The Immune Response to the Myxozoan Parasite *Myxobolus cerebralis* in Salmonids: A Review on Whirling Disease. Int. J. Mol. Sci..

[41] Saleh M., Montero R., Kumar G., Sudhagar A., Friedl A., Köllner B., El-Matbouli M. (2019). Kinetics of Local and Systemic Immune Cell Responses in Whirling Disease Infection and Resistance in Rainbow Trout. Parasites Vectors.

[42] Fernandes D.C., Eto S.F., Moraes A.C., Prado E.J.R., Medeiros A.S.R., Belo M.A.A., Samara S.I., Costa P.I., Pizauro J.M. (2019). Phagolysosomal Activity of Macrophages in Nile Tilapia (*Oreochromis niloticus*) Infected in Vitro by *Aeromonas hydrophila*: Infection and Immunotherapy. Fish Shellfish Immunol..

[43] Wang T., Secombes C.J. (2009). Identification and Expression Analysis of Two Fish-Specific IL-6 Cytokine Family Members, the Ciliary Neurotrophic Factor (CNTF)-like and M17 Genes, in Rainbow Trout *Oncorhynchus mykiss*. Mol. Immunol..

[44] Hanington P.C., Patten S.A., Reaume L.M., Waskiewicz A.J., Belosevic M., Ali D.W. (2008). Analysis of Leukemia Inhibitory Factor and Leukemia Inhibitory Factor Receptor in Embryonic and Adult Zebrafish (*Danio rerio*). Dev. Biol..

[45] Holzer A.S., Piazzon M.C., Barrett D., Bartholomew J.L., Sitjà-Bobadilla A. (2021). To React or Not to React: The Dilemma of Fish Immune Systems Facing Myxozoan Infections. Front. Immunol..

[46] Spagnoli S.T., Xue L., Murray K.N., Chow F., Kent M.L. (2015). *Pseudoloma neurophilia*: A Retrospective and Descriptive Study of Nervous System and Muscle Infections, with New Implications for Pathogenesis and Behavioral Phenotypes. Zebrafish.

[47] Chong R.S.-M. (2022). Whirling Disease. Aquaculture Pathophysiology.

[48] Saleh M., Hummel K., Schlosser S., Razzazi-Fazeli E., Bartholomew J.L., Holzer A., Secombes C.J., El-Matbouli M. (2024). The Myxozoans *Myxobolus cerebralis* and *Tetracapsuloides bryosalmonae* Modulate Rainbow Trout Immune Responses: Quantitative Shotgun Proteomics at the Portals of Entry after Single and Co-Infections. Front. Cell. Infect. Microbiol..

[49] Zhong L., Carvalho L.A., Gao S., Whyte S.K., Purcell S.L., Fast M.D., Cai W. (2023). Transcriptome Analysis Revealed Immune Responses in the Kidney of Atlantic Salmon (*Salmo salar*) Co-Infected with Sea Lice (*Lepeophtheirus salmonis*) and Infectious Salmon Anemia Virus. Fish Shellfish Immunol..

[50] Cai W., Kumar S., Navaneethaiyer U., Caballero-Solares A., Carvalho L.A., Whyte S.K., Purcell S.L., Gagne N., Hori T.S., Allen M. (2022). Transcriptome Analysis of Atlantic Salmon (*Salmo salar*) Skin in Response to Sea Lice and Infectious Salmon Anemia Virus Co-Infection Under Different Experimental Functional Diets. Front. Immunol..

[51] Chan J.T.H., Picard-Sánchez A., Majstorović J., Rebl A., Koczan D., Dyčka F., Holzer A.S., Korytář T. (2023). Red Blood Cells in Proliferative Kidney Disease—Rainbow Trout (*Oncorhynchus mykiss*) Infected by *Tetracapsuloides bryosalmonae* Harbor IgM+ Red Blood Cells. Front. Immunol..

[52] Saleh M., Hummel K., Schlosser S., Razzazi-Fazeli E., Bartholomew J.L., Secombes C.J., El-Matbouli M. (2026). Quantitative Proteomic Profiling of Stress and Immunological Responses in Target Organs Reveals How Myxozoan Parasites Determine the Outcome of Rainbow Trout Co-Infections. Arch. Microbiol. Immunol..

[53] Soliman A.M., Barreda D.R. (2023). The Acute Inflammatory Response of Teleost Fish. Dev. Comp. Immunol..

[54] Prado L.G., Camara N.O.S., Barbosa A.S. (2023). Cell Lipid Biology in Infections: An Overview. Front. Cell. Infect. Microbiol..

[55] Liu X., Gu B., Zhu D., Tang X., Zhan Y. (2024). Metabolomics and Lipidomics Profiling Reveal Distinctive Responses of Olive Flounder (*Paralichthys olivaceus*) to Bacterial and Viral Infections. Aquaculture.

[56] Stolte E.H., Nabuurs S.B., Bury N.R., Sturm A., Flik G., Savelkoul H.F.J., Lidy Verburg-van Kemenade B.M. (2008). Stress and Innate Immunity in Carp: Corticosteroid Receptors and pro-Inflammatory Cytokines. Mol. Immunol..

[57] Faught E., Schaaf M.J.M. (2023). The Mineralocorticoid Receptor Plays a Crucial Role in Macrophage Development and Function. Endocrinology.

[58] Hartigan A., Kosakyan A., Pecková H., Eszterbauer E., Holzer A.S. (2020). Transcriptome of *Sphaerospora molnari* (Cnidaria, Myxosporea) Blood Stages Provides Proteolytic Arsenal as Potential Therapeutic Targets against Sphaerosporosis in Common Carp. BMC Genom..

[59] Shivam S., El-Matbouli M., Kumar G. (2021). Kinetics of Parasite-Specific Antibody and B-Cell-Associated Gene Expression in Brown Trout, *Salmo trutta* during Proliferative Kidney Disease. Biology.

[60] Deal C.K., Volkoff H. (2020). The Role of the Thyroid Axis in Fish. Front. Endocrinol..

[61] Reite O.B., Evensen Ø. (2006). Inflammatory Cells of Teleostean Fish: A Review Focusing on Mast Cells/Eosinophilic Granule Cells and Rodlet Cells. Fish Shellfish Immunol..

[62] Balla K.M., Lugo-Villarino G., Spitsbergen J.M., Stachura D.L., Hu Y., Bañuelos K., Romo-Fewell O., Aroian R.V., Traver D. (2010). Eosinophils in the Zebrafish: Prospective Isolation, Characterization, and Eosinophilia Induction by Helminth Determinants. Blood.

[63] Evensen Ø., Brudeseth B., Mutoloki S. (2005). Inflammatory Processes in Fish. Developments in Biologicals.

[64] Bailey C., Holland J.W., Secombes C.J., Tafalla C. (2020). A Portrait of the Immune Response to Proliferative Kidney Disease (PKD) in Rainbow Trout. Parasite Immunol..

[65] Secombes C.J., Wang T., Bird S. (2011). The Interleukins of Fish. Dev. Comp. Immunol..

[66] Uzé G., Monneron D. (2007). IL-28 and IL-29: Newcomers to the Interferon Family. Biochimie.

[67] Zwollo P., Haines A., Rosato P., Gumulak-Smith J. (2008). Molecular and Cellular Analysis of B-Cell Populations in the Rainbow Trout Using Pax5 and Immunoglobulin Markers. Dev. Comp. Immunol..

[68] Abos B., Estensoro I., Perdiguero P., Faber M., Hu Y., Rosales P.D., Granja A.G., Secombes C.J., Holland J.W., Tafalla C. (2018). Dysregulation of B Cell Activity during Proliferative Kidney Disease in Rainbow Trout. Front. Immunol..

[69] Qiu Y., Yin Y., Ruan Z., Gao Y., Bian C., Chen J., Wang X., Pan X., Yang J., Shi Q. (2020). Comprehensive Transcriptional Changes in the Liver of Kanglang White Minnow (*Anabarilius grahami*) in Response to the Infection of Parasite *Ichthyophthirius multifiliis*. Animals.

[70] Quesada-García A., Valdehita A., Kropf C., Casanova-Nakayama A., Segner H., Navas J.M. (2014). Thyroid Signaling in Immune Organs and Cells of the Teleost Fish Rainbow Trout (*Oncorhynchus mykiss*). Fish Shellfish Immunol..

[71] Aedo J.E., Zuloaga R., Aravena-Canales D., Molina A., Valdés J.A. (2023). Role of Glucocorticoid and Mineralocorticoid Receptors in Rainbow Trout (*Oncorhynchus mykiss*) Skeletal Muscle: A Transcriptomic Perspective of Cortisol Action. Front. Physiol..

[72] Saleh M., Friedl A., Srivastava M., Soliman H., Secombes C.J., El-Matbouli M. (2020). STAT3/SOCS3 Axis Contributes to the Outcome of Salmonid Whirling Disease. PLoS ONE.

[73] Sudhagar A., Ertl R., Kumar G., El-Matbouli M. (2019). Transcriptome Profiling of Posterior Kidney of Brown Trout, *Salmo trutta*, during Proliferative Kidney Disease. Parasites Vectors.

[74] Król E., Douglas A., Tocher D.R., Crampton V.O., Speakman J.R., Secombes C.J., Martin S.A.M. (2016). Differential Responses of the Gut Transcriptome to Plant Protein Diets in Farmed Atlantic Salmon. BMC Genom..

[75] Clinton M., Król E., Sepúlveda D., Andersen N.R., Brierley A.S., Ferrier D.E.K., Hansen P.J., Lorenzen N., Martin S.A.M. (2021). Gill Transcriptomic Responses to Toxin-Producing Alga Prymnesium Parvum in Rainbow Trout. Front. Immunol..

[76] American Veterinary Medical Association (2020). AVMA Guidelines for the Euthanasia of Animals: 2020 Edition.

[77] Manual of Diagnostic Tests for Aquatic Animals (2021) Diseases of Fish: General Information. https://www.woah.org/fileadmin/Home/eng/Health_standards/aahm/current/2.3.00_INTRO_FISH.pdf.

[78] Kelley G., Zagmutt-Vergara F., Leutenegger C., Adkison M., Baxa D., Hedrick R. (2004). Identification of a Serine Protease Gene Expressed by *Myxobolus cerebralis* during Development in Rainbow Trout *Oncorhynchus mykiss*. Dis. Aquat. Org..

[79] Gorgoglione B., Wang T., Secombes C.J., Holland J.W. (2013). Immune Gene Expression Profiling of Proliferative Kidney Disease in Rainbow Trout *Oncorhynchus mykiss* Reveals a Dominance of Anti-Inflammatory, Antibody and T Helper Cell-like Activities. Vet. Res..

[80] Kumar G., Abd-Elfattah A., El-Matbouli M. (2014). Differential Modulation of Host Genes in the Kidney of Brown Trout *Salmo trutta* during Sporogenesis of *Tetracapsuloides bryosalmonae* (Myxozoa). Vet. Res..

[81] Bolger A.M., Lohse M., Usadel B. (2014). Trimmomatic: A Flexible Trimmer for Illumina Sequence Data. Bioinformatics.

[82] Raudvere U., Kolberg L., Kuzmin I., Arak T., Adler P., Peterson H., Vilo J. (2019). G:Profiler: A Web Server for Functional Enrichment Analysis and Conversions of Gene Lists (2019 Update). Nucleic Acids Res..

[83] Ge S.X., Jung D., Yao R. (2020). ShinyGO: A Graphical Gene-Set Enrichment Tool for Animals and Plants. Bioinformatics.

[84] Coulibaly I., Gahr S.A., Palti Y., Yao J., Rexroad C.E. (2006). Genomic Structure and Expression of Uncoupling Protein 2 Genes in Rainbow Trout (*Oncorhynchus mykiss*). BMC Genom..

[85] Baerwald M.R. (2013). Temporal expression patterns of rainbow trout immune-related genes in response to Myxobolus cerebralis exposure. Fish Shellfish Immunol..

[86] Maisey K., Montero R., Corripio-Miyar Y., Toro-Ascuy D., Valenzuela B., Reyes-Cerpa S., Sandino A.M., Zou J., Wang T., Secombes C.J. (2016). Isolation and characterization of salmonid CD4+ T cells. J. Immunol..

