# Supplementary material for: Tissue-Specific Transcriptomic Insights into Myxozoan Infections: Immune and Stress Responses in the Kidney and Head Cartilage of Rainbow Trout
Source: Int J Mol Sci. 2026 Aug 14;27(16):7253. doi: 10.3390/ijms27167253 (PMC13512926; doi:10.3390/ijms27167253)
Supplement: Supplementary file 1 [file ijms-27-07253-s001.zip › Supplementary file S6.pdf]

Supplementary Table S1. Significantly regulated KEGG pathways in infected kidney.

Sr No.	Pathways	Group	Fold Enrichment	Pathway ID	No. of identified genes	Genes
**Upregulated Pathways**						
1	Progesterone-mediated oocyte maturation	Mc	12.82	hsa04914	4	*mos, mapk12, ccna1, ccnb1*
2	Cytokine-cytokine receptor interaction	Mc^{+}	9.76	hsa04060	34	*ccl26, ccr1, ccr3, ccr4, ccr5, ccr8, csf1r, cx3cr1, tnfrsf13b, ifnar2, ifng, ifngr1, il2rb, il2rg, il3ra, il10, il10rb, il13ra2, tnfrsf9, ackr4, ccl1, ccl2, ccl7, ccl8, ccl11, ccl13, ccl22, ccl24, cx3cl1, ccr2, il1r2, tnfsf14, tnfrsf6b, cd4*
3	Th17 cell differentiation	Mc^{+}	7.82	hsa04659	10	*hsp90aa1, ifngr1, il2rb, il2rg, irf4, jak1, ppp3ca, stat3, cd4*
4	T cell receptor signaling pathway	Tb	5.87	hsa04660	11	*rasgrp1, akt1, fyn, ifng, il10, lcp2, nfatc2, ppp3ca, cd4, cd8b, cd8b2*
5	Antigen processing and presentation	Tb	5.64	hsa04612	8	*pdia3, hspa2, hspa5, hspa8, ifng, cd4, cd8b, cd8b2*
6	JAK-STAT signaling pathway	Tb^{+}	4.89	hsa04630	9	*socs4, ifnar2, ifng, ifngr1, il2rb, il2rg, il3ra, il10, il13ra2*
7	TNF signaling pathway	Tb^{+}	4.71	hsa04668	6	*birc2, birc3, irf1, ccl2, cx3cl1, traf1*

Downregulated Pathways						
8	Biosynthesis of amino acids	Mc	15.66	hsa01230	4	*eno3, aldoa, pfkm, pgam2*
9	HIF-1 signaling pathway	Mc	7.97	hsa04066	3	*eno3, aldoa, pfkm*
10	Mineral absorption	Mc^{+}	14.9	hsa04978	10	*mt1a, mt1b, mt1e, mt1f, mt1g, mt1h, mt1m, mt1x, mt2a, mt1hl1*
11	Vitamin digestion and absorption	Mc^{+}	11.17	hsa04977	3	*apoa1, apob, tcn2*
12	Ascorbate and aldarate metabolism	Tb	21	hsa00053	19	*ugt2b11, ugt2a1, aldh1b1, ugt2b28, ugt1a10, ugt1a8, ugt1a7, ugt1a6, ugt1a5, ugt1a9, ugt1a4, ugt1a1, ugt1a3, miox, ugt2a2, ugt2b4, ugt2b7, ugt2b17, rgn*
13	Fructose and mannose metabolism	Tb	6.03	hsa00051	6	*aldoa, khk, pfkl, pfkm, akr1b10, fbp2*
14	Nitrogen metabolism	Tb^{+}	37.21	hsa00910	6	*ca13, ca1, ca2, ca3, ca5a, ca9*
15	Mineral absorption	Tb^{+}	19.33	hsa04978	11	*mt1a, mt1b, mt1e, mt1f, mt1g, mt1h, mt1m, mt1x, mt2a, atp1a4, mt1hl1*
