# Supplementary material for: Tissue-Specific Transcriptomic Insights into Myxozoan Infections: Immune and Stress Responses in the Kidney and Head Cartilage of Rainbow Trout
Source: Int J Mol Sci. 2026 Aug 14;27(16):7253. doi: 10.3390/ijms27167253 (PMC13512926; doi:10.3390/ijms27167253)
Supplement: Supplementary file 1 [file ijms-27-07253-s001.zip › Supplementary file S7.pdf]

Supplementary Table S2. Significantly regulated KEGG pathways in infected head cartilage.

Sr No.	Pathways	Group	Fold Enrichment	Pathway ID	No. of identified genes	Genes
**Upregulated Pathways**						
1	JAK-STAT signaling pathway	Mc	12.52	hsa04630	7	*il23r, il2rg, il3ra, il6r, il12rb1, crlf2, osmr*
2	PPAR signaling pathway	Mc	11.59	hsa03320	3	*gk, gk2, mmp1*
3	Glycosphingolipid biosynthesis-globo and isoglobo series	Mc^+	43.58	hsa00603	2	*gbgt1, st3gal1*
4	Fc gamma R-mediated phagocytosis	Mc^+	10.11	hsa04666	3	*dnm2, ncf1, pla2g4c*
5	VEGF signaling pathway	Tb	29.83	hsa04370	1	*pla2g4c*
6	Inflammatory mediator regulation of TRP channels	Tb	17.96	hsa04750	1	*pla2g4c*

7	Phagosome	Tb⁺	14.21	hsa04145	12	*clec4m, ctsl, cybb, fcgr1a, fcgr2a, fcgr2b, fcgr3a, fcgr3b, cd209, itgb2, mrc1, ncf1*
8	Fc gamma R-mediated phagocytosis	Tb⁺	12.90	hsa04666	7	*fcgr1a, fcgr2a, fcgr2b, fcgr3a, fcgr3b, ncf1, syk*
**Downregulated Pathways**						
9	Cell adhesion molecules	Mc	8.50	hsa04514	4	*cd226, l1cam, mag, nrcam*
10	Tryptophan metabolism	Mc	15.79	hsa00380	2	*asmt, tph1*
11	Mineral absorption	Mc⁺	18.83	hsa04978	4	*clcn2, atp1a1, slc26a6, slc8a2*
12	Primary immunodeficiency	Mc⁺	14.87	hsa05340	2	*rag1, cd8a*
13	Viral protein interaction with cytokine and cytokine receptor	Tb	7.46	hsa04061	1	*il10rb*
14	Metabolic pathways	Tb	2.88	hsa01100	6	*b3gat1, hmgcs1, asmt, rdh8, ptgds, tph1*
15	Mineral absorption	Tb⁺	11.30	hsa04978	4	*clcn2, atp1a1, slc26a6, slc8a2*
