# Supplementary material for: Tissue-Specific Transcriptomic Insights into Myxozoan Infections: Immune and Stress Responses in the Kidney and Head Cartilage of Rainbow Trout
Source: Int J Mol Sci. 2026 Aug 14;27(16):7253. doi: 10.3390/ijms27167253 (PMC13512926; doi:10.3390/ijms27167253)
Supplement: Supplementary file 1 [file ijms-27-07253-s001.zip › Supplementary file S8.pdf]

Table S3. Immune and metabolic pathways enriched in the kidney and head cartilage of rainbow trout during single and co-infection with *M. cerebralis* and *T. bryosalmonae*.

Organ/Regulation	Mc	Mc^{+}	Tb	Tb^{+}
**Kidney (upregulated)**	Macrophage activation	Macrophage activation	Macrophage activation (M1-like)	Macrophage- & eosinophil-mediated inflammation
	NK cell cytotoxicity	Plasma cell differentiation	Chronic inflammatory response	T-cell receptor signaling
	Mast cell differentiation	IL-2, IL-15 signaling	IL-*8* mediated chemotaxis	Plasma cell differentiation
				IL-2 signaling
**Kidney (Downregulated)**	Eosinophil-related genes	Th17, IL-23 signaling	Th17, IL-23 signaling	IL-23, Th17, IL-12 signaling
	Chemokine signaling	MHC-related pathways	Succinate dehydrogenase	TLR & death receptor activity
			Xenobiotic metabolism	
**Head cartilage (Upregulated)**	Th17 and IL-23 signaling	Phagolysosome processes	Lipid metabolism	IL-23, Th17 signaling

	Phagolysosome	ECM remodeling	Corticosteroid signaling	FcγRIII activity
	IL-6 family cytokines	Metabolic activation	Metalloaminopeptidase activity	Arachidonate 8(S)-lipoxygenase
**Head cartilage (Downregulated)**	Neural development	Epithelial integrity	B-cell development	Developmental processes
	Cytoskeletal organization	Lymphocyte differentiation	Antigen presentation	
