# Supplementary material for: Tissue-Specific Transcriptomic Insights into Myxozoan Infections: Immune and Stress Responses in the Kidney and Head Cartilage of Rainbow Trout
Source: Int J Mol Sci. 2026 Aug 14;27(16):7253. doi: 10.3390/ijms27167253 (PMC13512926; doi:10.3390/ijms27167253)
Supplement: Supplementary file 1 [file ijms-27-07253-s001.zip › Supplementary file S9.pdf]

Gene	GenBank accession number	Forward primer (5′ to 3′)	Reverse primer (5′ to 3′)	Amplicon size (bp)	References
IL-17A	100136642	CGTGTCGAAGTACCTGGTTGTGT	GGTTCTCCACTGTAGTGCTTTTCCA	212	Gorgoglione et al. 2013
SOCS-3	100272212	CACAGAGAAACCGTTAAAAGGACTATCC	AAGGGGCTGCTGCTCATGAC	228	Gorgoglione et al. 2013
STAT-3	100136756	GAATGAAGGGTATATTCTGG	TCCCACTGATGTCCTTTTCC	152	Baerwald et. Al 2013
STAT-1	100136755	GACCAGCGAACCCAAGAACCTGAA	CACAAAGCCCAGGATGCAACCAT	319	Maisey et al. 2016

References

Baerwald, M. R. (2013). Temporal expression patterns of rainbow trout immune-related genes in response to Myxobolus cerebralis exposure. *Fish & shellfish immunology*, *35*(3), 965-971.

Gorgoglione, B., Wang, T., Secombes, C. J., & Holland, J. W. (2013). Immune gene expression profiling of Proliferative Kidney Disease in rainbow trout Oncorhynchus mykiss reveals a dominance of anti-inflammatory, antibody and T helper cell-like activities. *Veterinary Research*, *44*(1), 55.

Maisey, K., Montero, R., Corripio-Miyar, Y., Toro-Ascuy, D., Valenzuela, B., Reyes-Cerpa, S., ... & Imarai, M. (2016). Isolation and characterization of salmonid CD4+ T cells. *The Journal of Immunology*, *196*(10), 4150-4163.
